# Supplementary material for: Computer-Aided Design and Synthesis of (Functionalized quinazoline)–(α-substituted coumarin)–arylsulfonate Conjugates against Chikungunya Virus
Source: Int J Mol Sci. 2022 Jul 11;23(14):7646. doi: 10.3390/ijms23147646 (PMC9322071; doi:10.3390/ijms23147646)
Supplement: Supplementary file 1 [file ijms-23-07646-s001.zip › ijms-1773329-supplementary.pdf]

Revised. II

Computer-aided design and synthesis of (functionalized quinazoline)–(α-substituted coumarin)–arylsulfonate conjugates against chikungunya virus

Jih Ru Hwu,[1,2,*] Animesh Roy,[1] Shwu-Chen Tsay,[1,2] Wen-Chieh Huang,[1,2] Chun-Cheng Lin,[1,2] Kuo Chu Hwang,[1,2] Yu-Chen Hu,[2,3] Fa-Kuen Shieh,[4] Pieter Leyssen,[5] Johan Neyts[5]

[1] *Department of Chemistry, National Tsing Hua University, Hsinchu 300044, Taiwan*

[2] *Frontier Research Center on Fundamental and Applied Sciences of Matters, National Tsing Hua University, Hsinchu 300044, Taiwan*

[3]*Department of Chemical Engineering, National Tsing Hua University, Hsinchu 300044, Taiwan*

[4]*Department of Chemistry, National Central University, Jhongli City 320317, Taiwan*

[5]*Rega Institute for Medical Research, Katholieke Universiteit Leuven, Minderbroedersstraat 10, B-3000 Leuven, Belgium*

*Corresponding Author

E-mail addresses: jrhwu@mx.nthu.edu.tw (J.R. Hwu)

^{1}H NMR, ^{13}C NMR, and IR Spectra of New Compounds

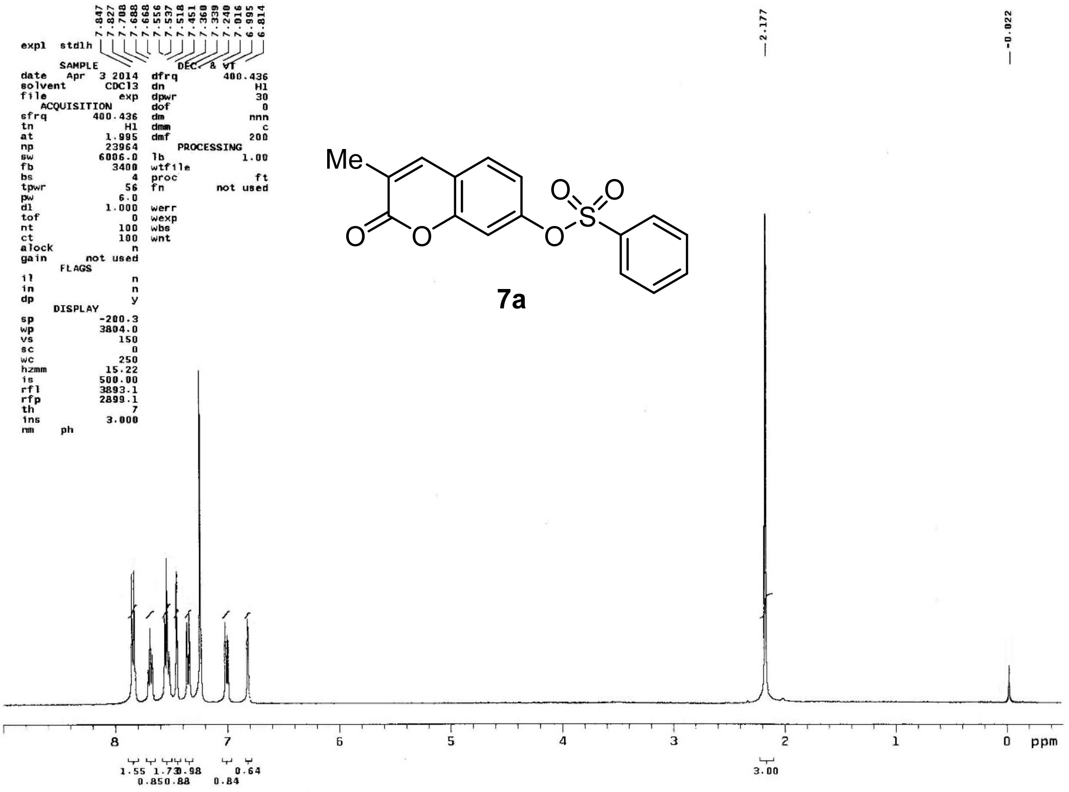

^{1}H NMR spectrum of compound **7a**

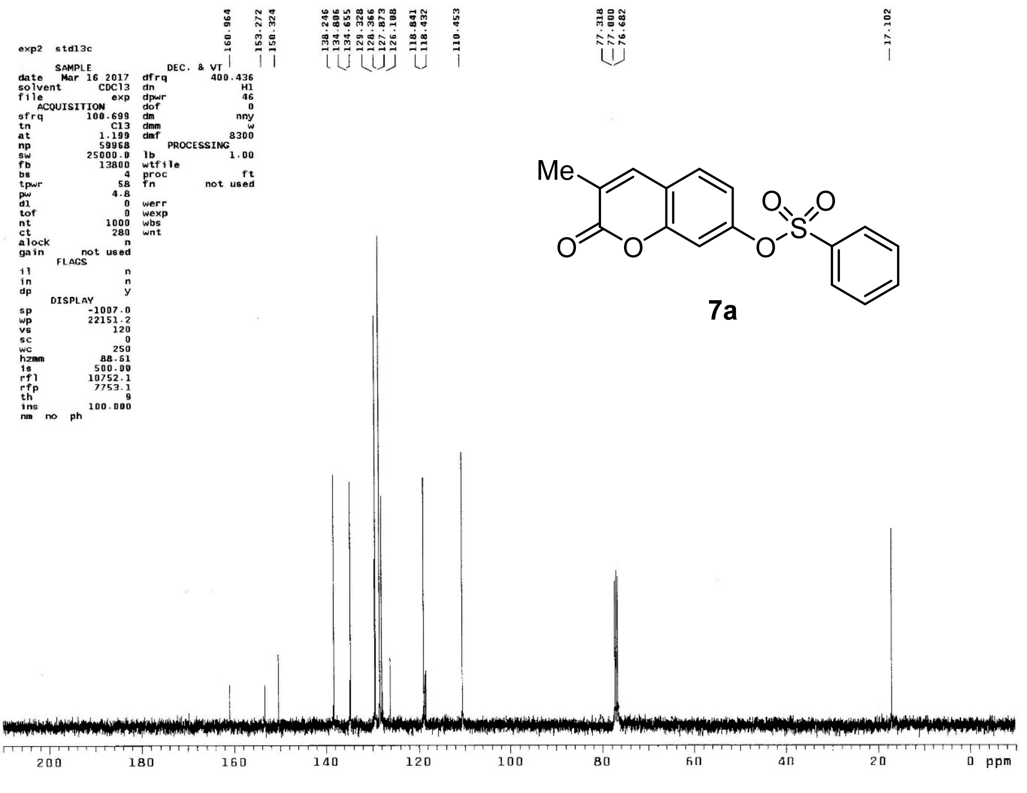

^{13}C NMR spectrum of compound **7a**

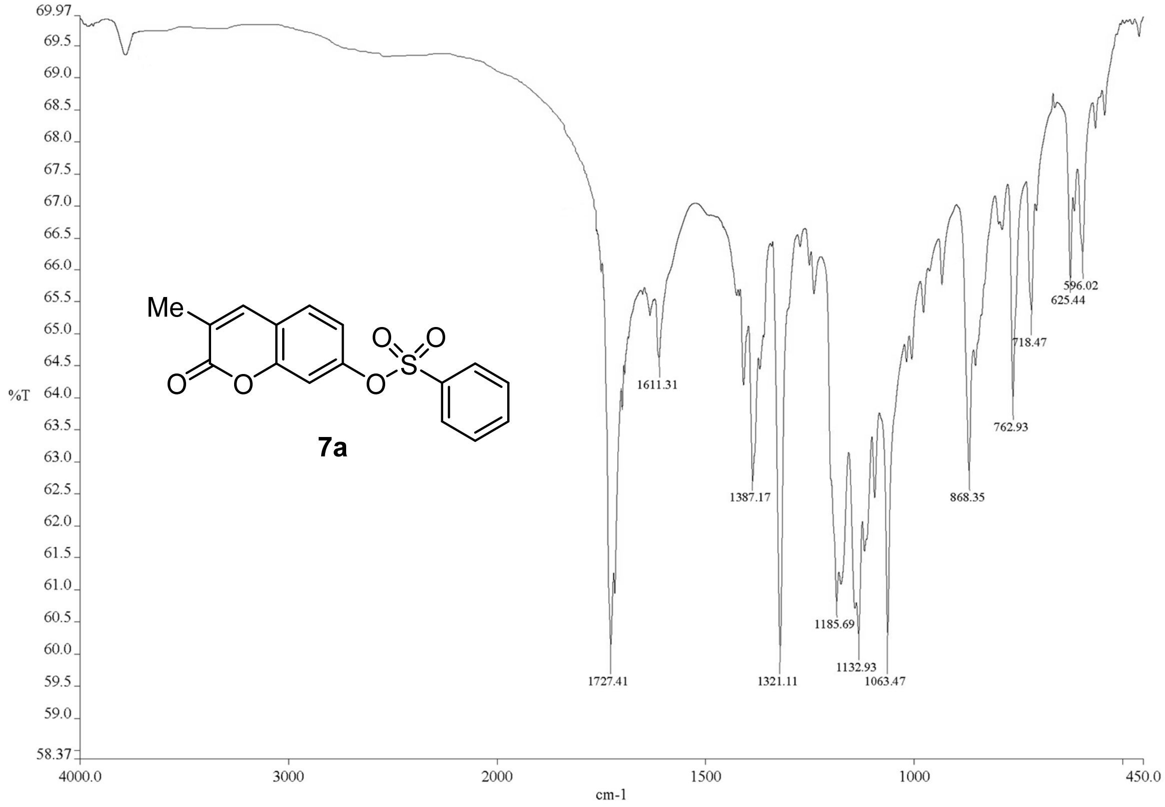

IR spectrum of compound **7a**

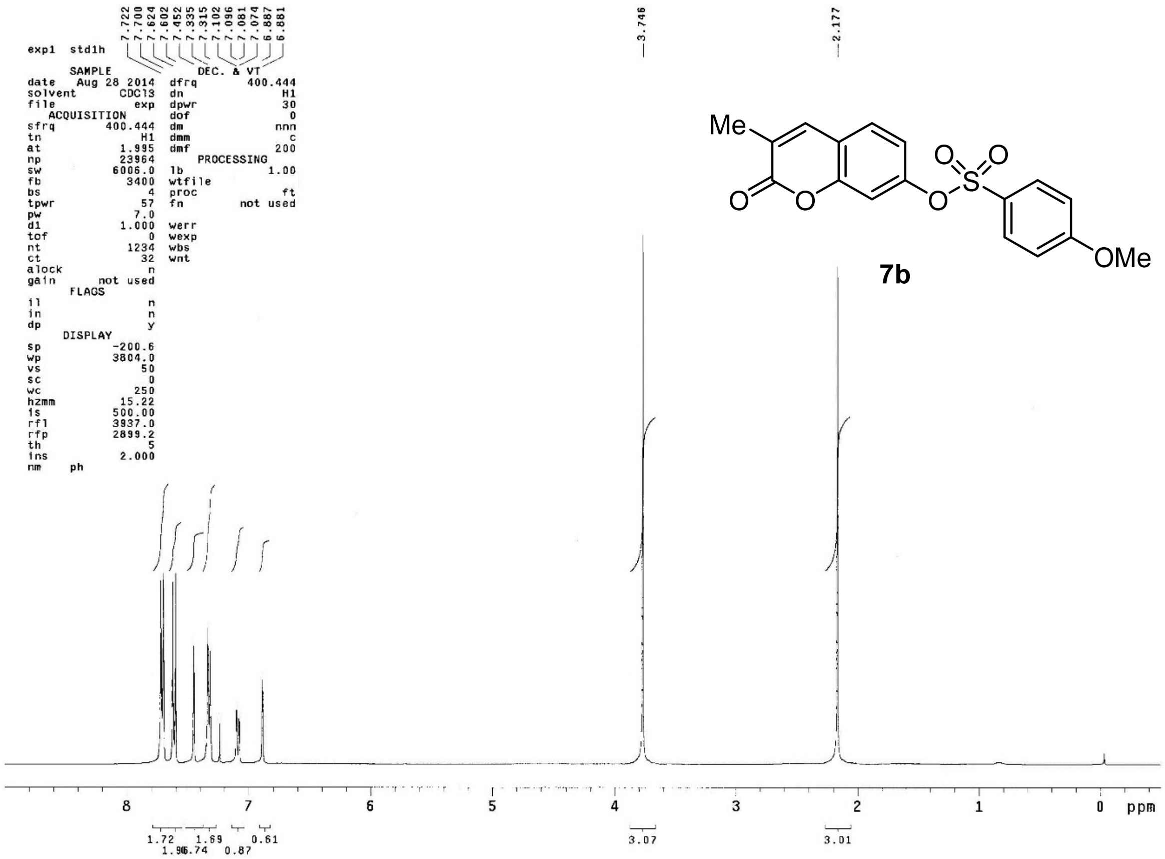

^{1}H NMR spectrum of compound **7b**

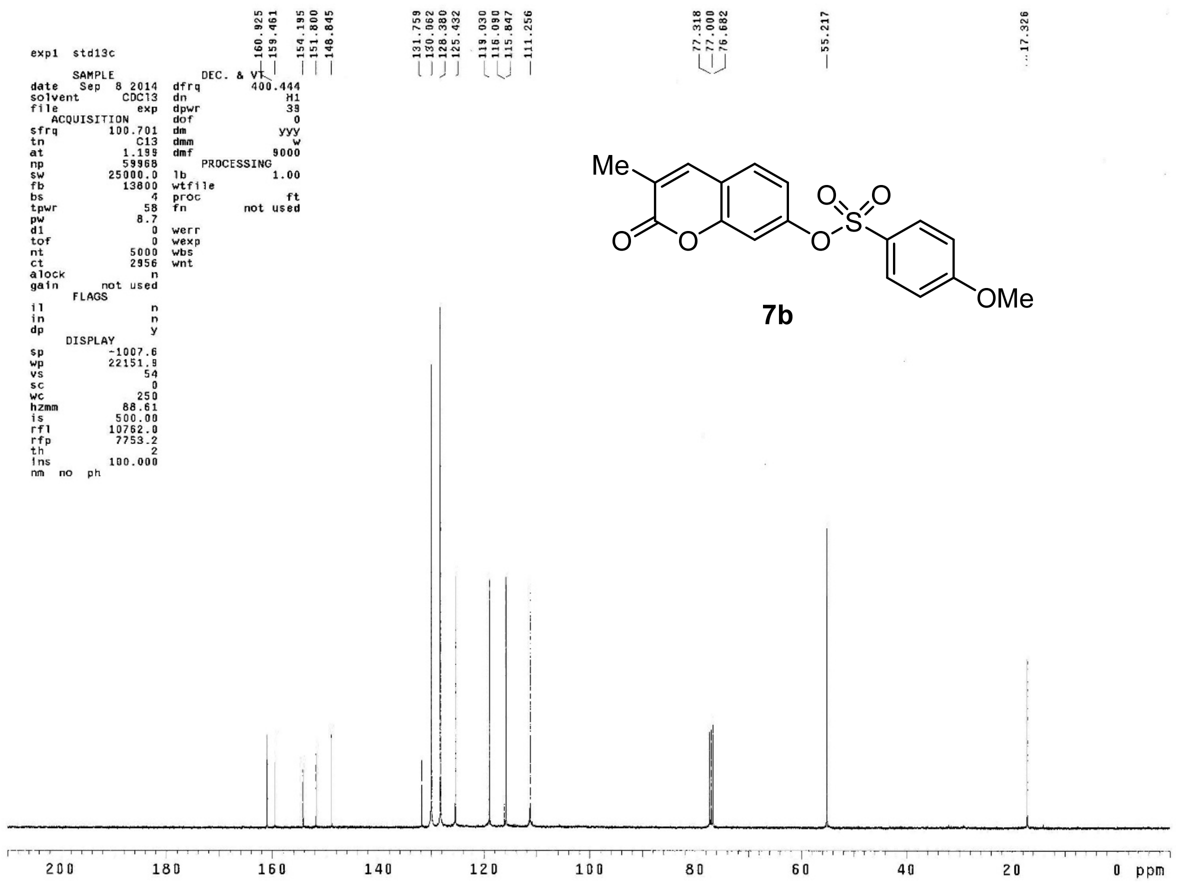

^{13}C NMR spectrum of compound **7b**

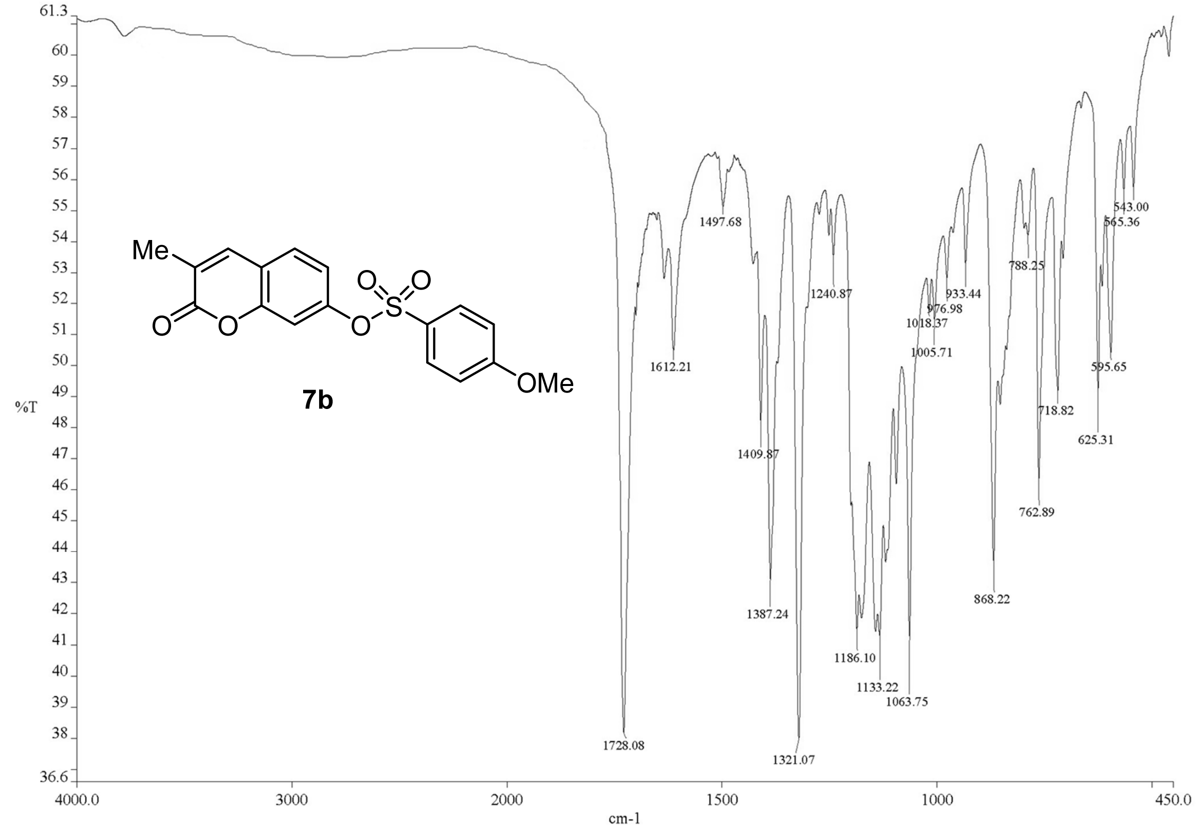

IR spectrum of compound **7b**

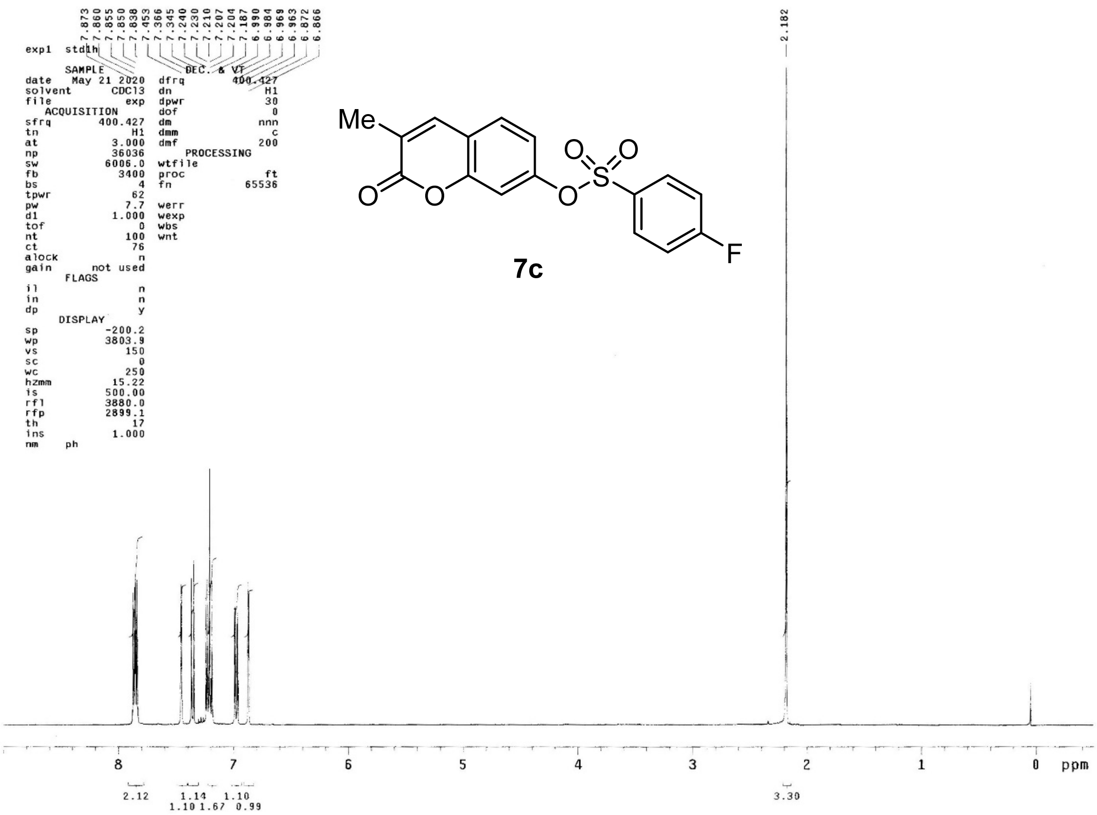

^{1}H NMR spectrum of compound **7c**

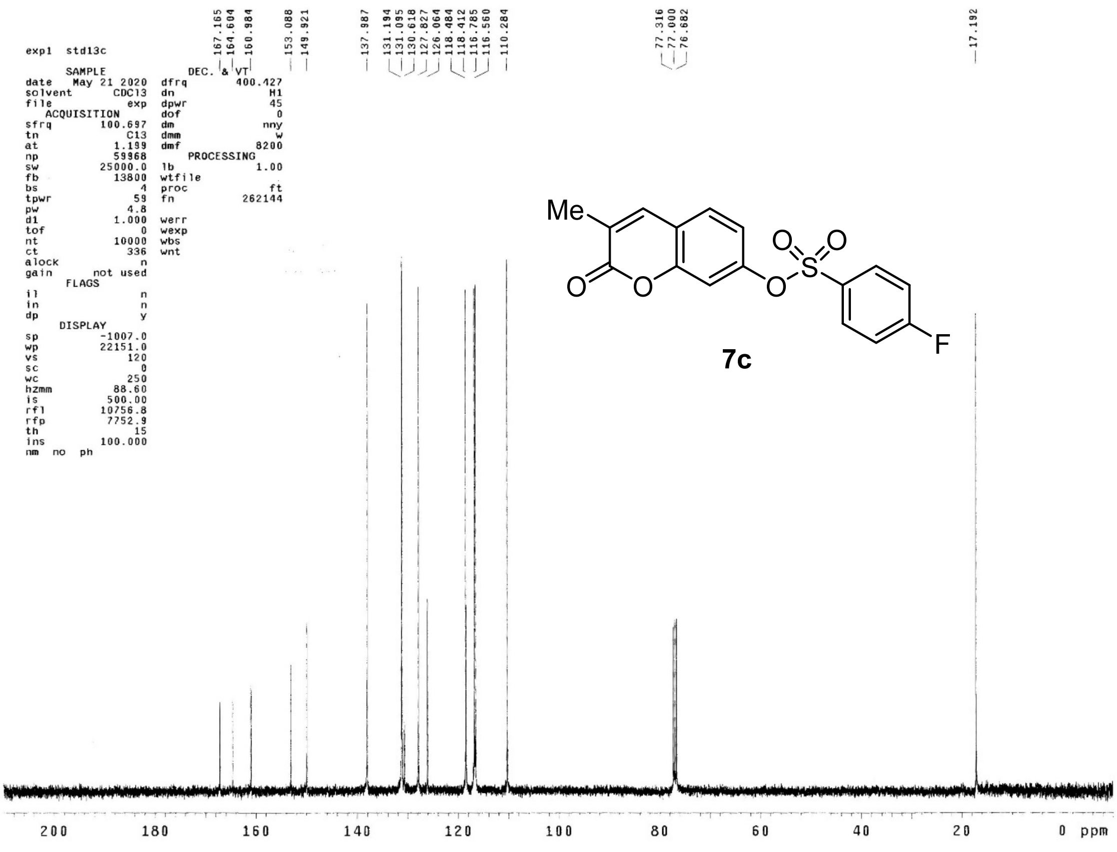

^{13}C NMR spectrum of compound **7c**

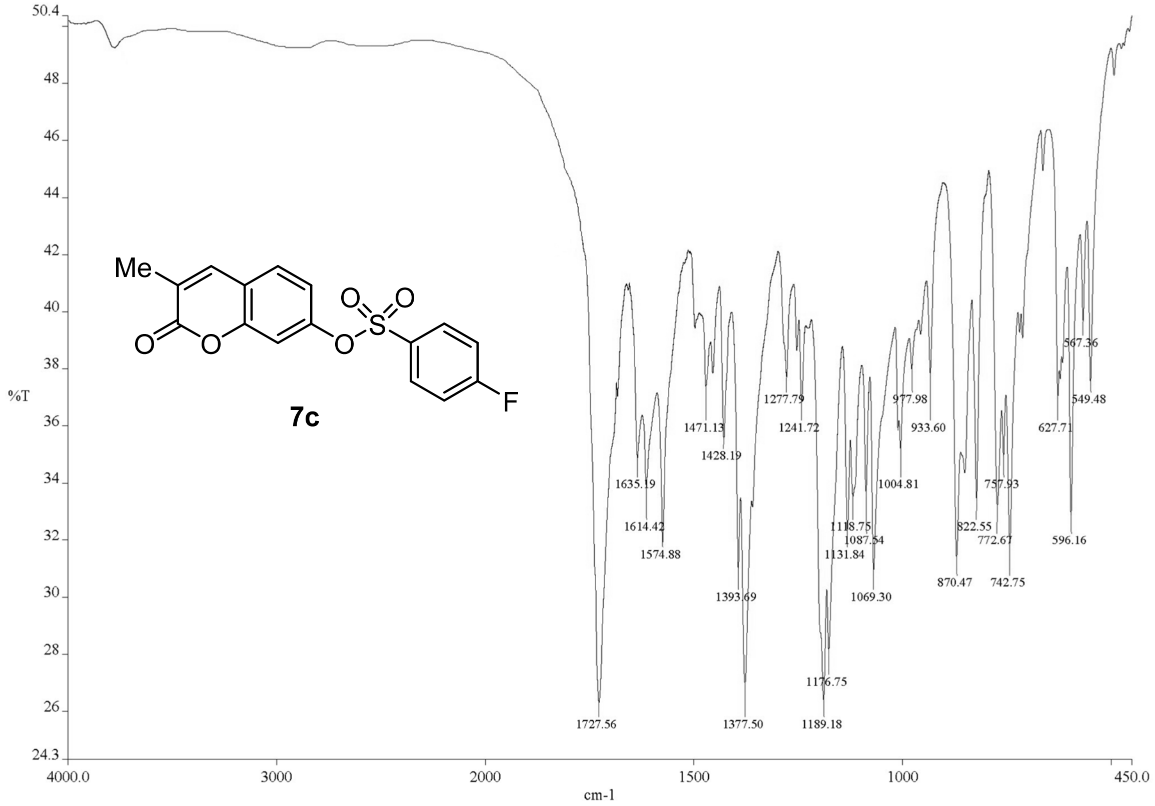

IR spectrum of compound **7c**

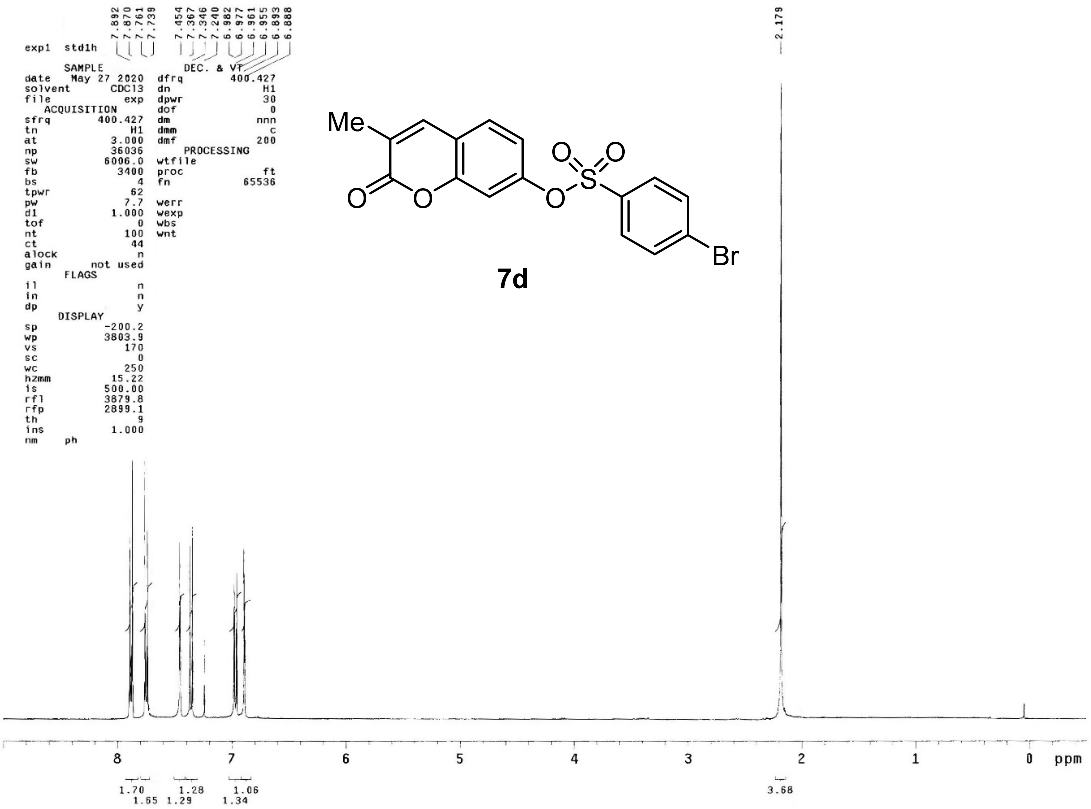

^{1}H NMR spectrum of compound **7d**

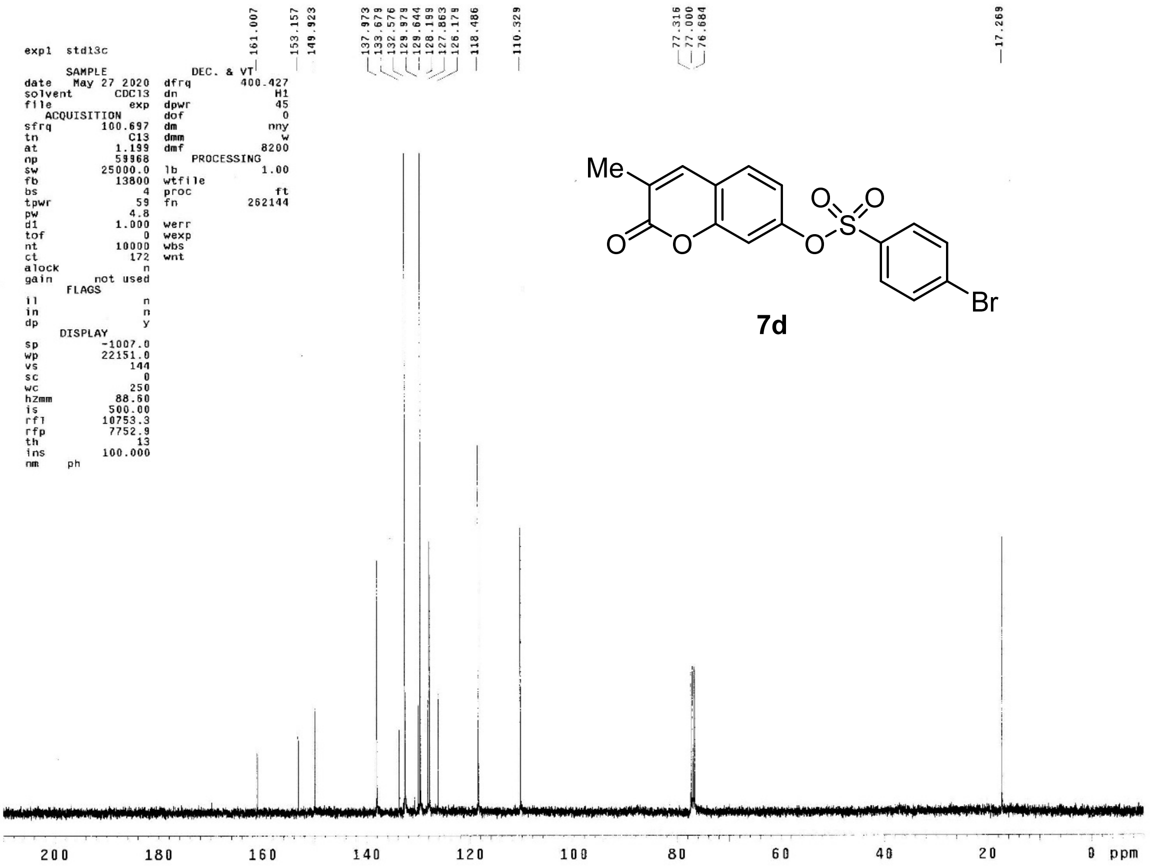

^{13}C NMR spectrum of compound **7d**

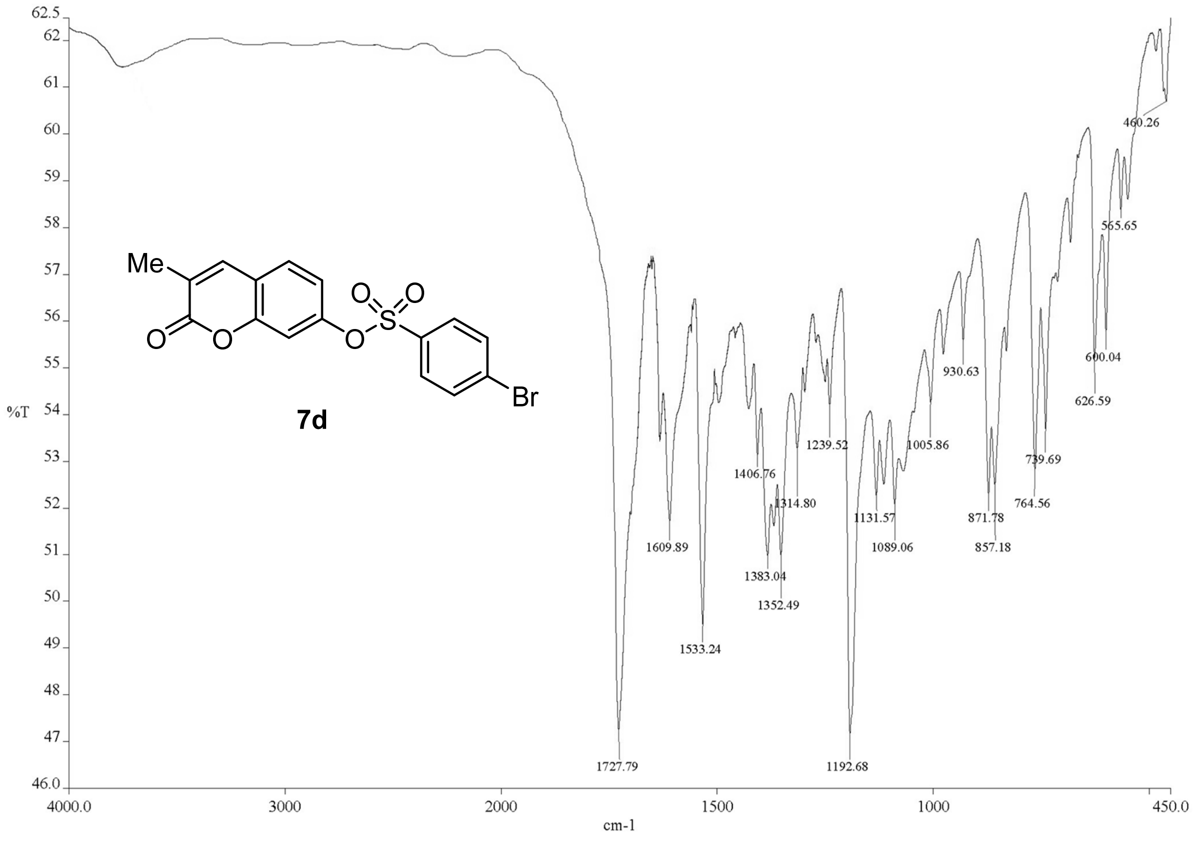

IR spectrum of compound **7d**

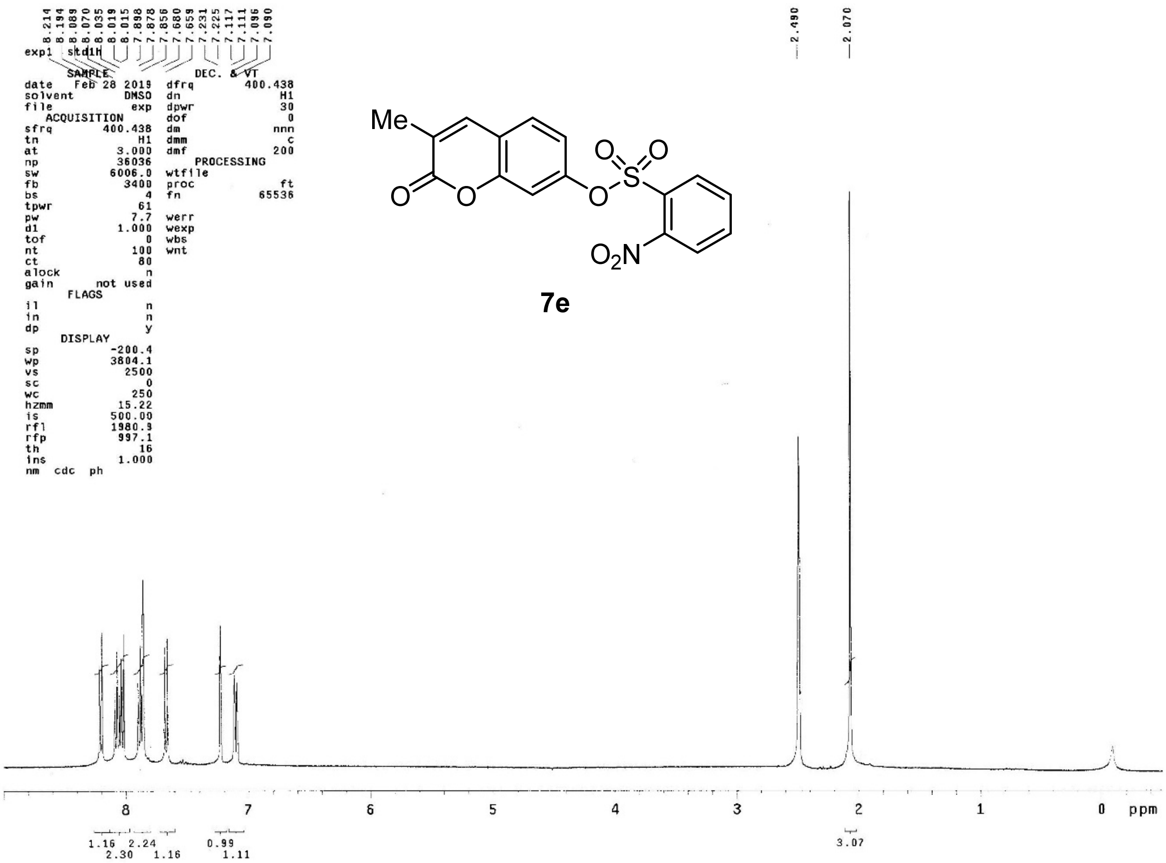

^{1}H NMR spectrum of compound **7e**

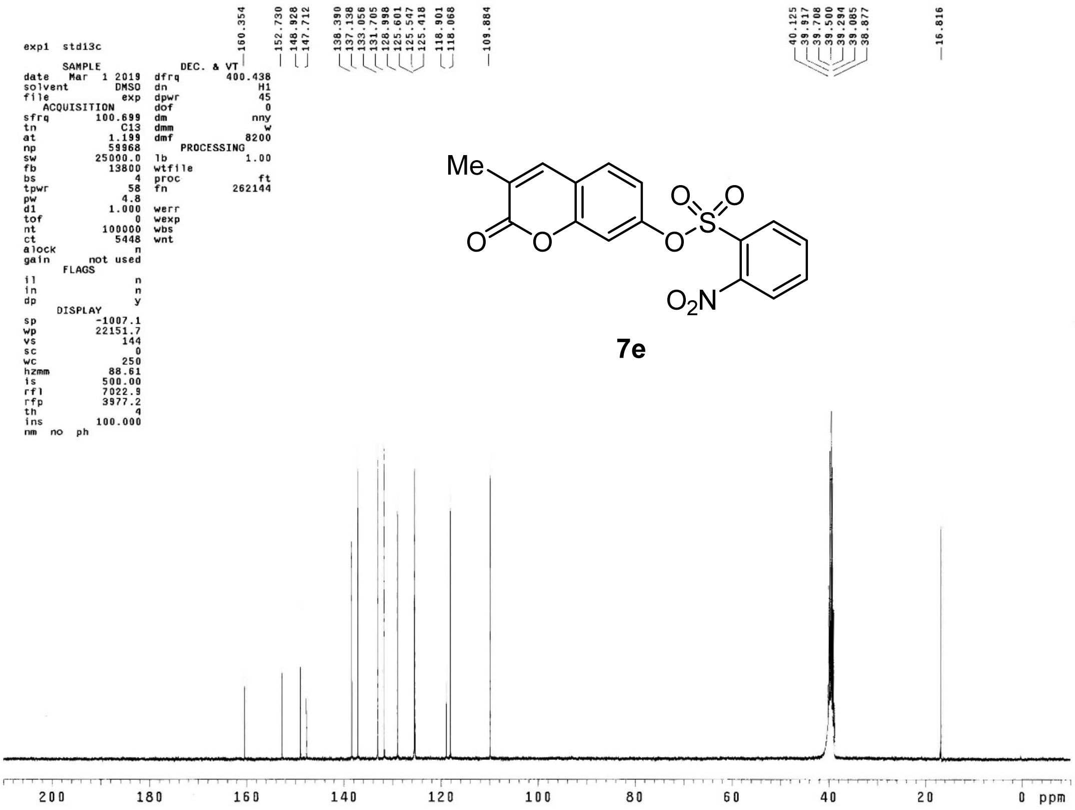

^{13}C NMR spectrum of compound **7e**

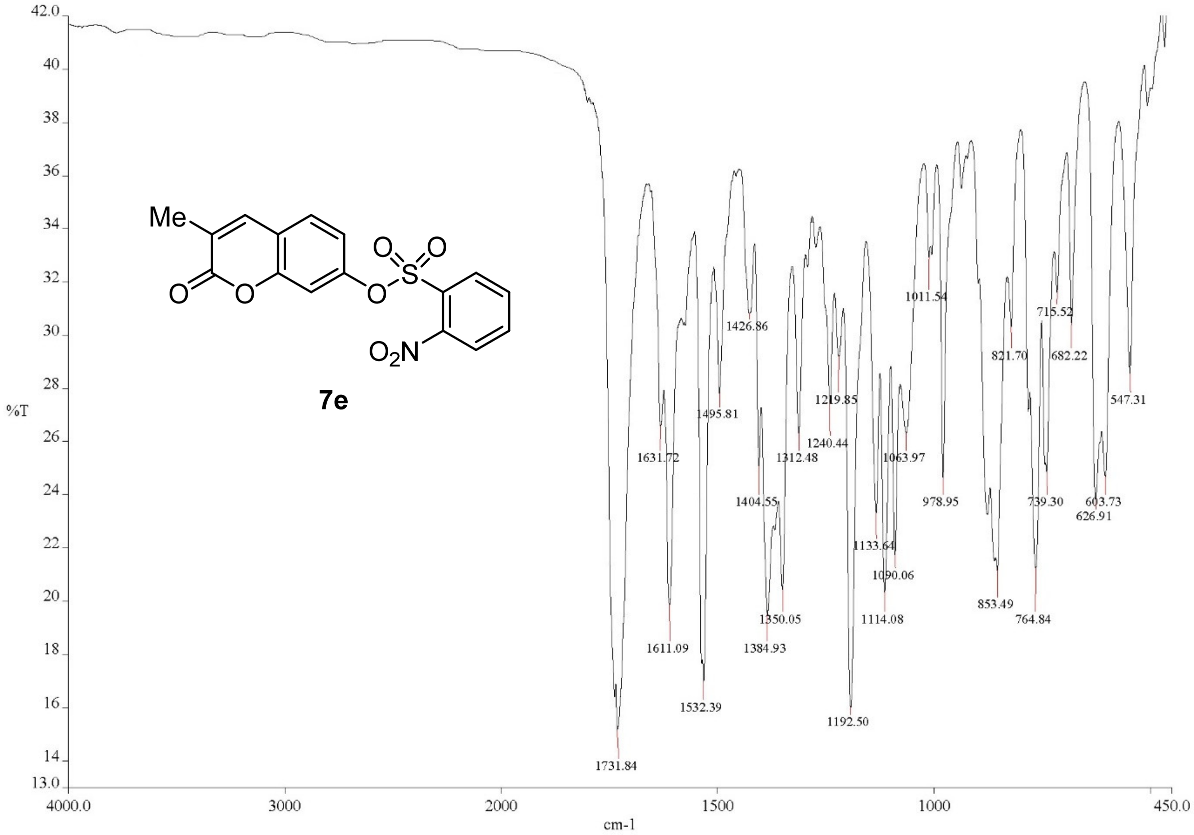

IR spectrum of compound **7e**

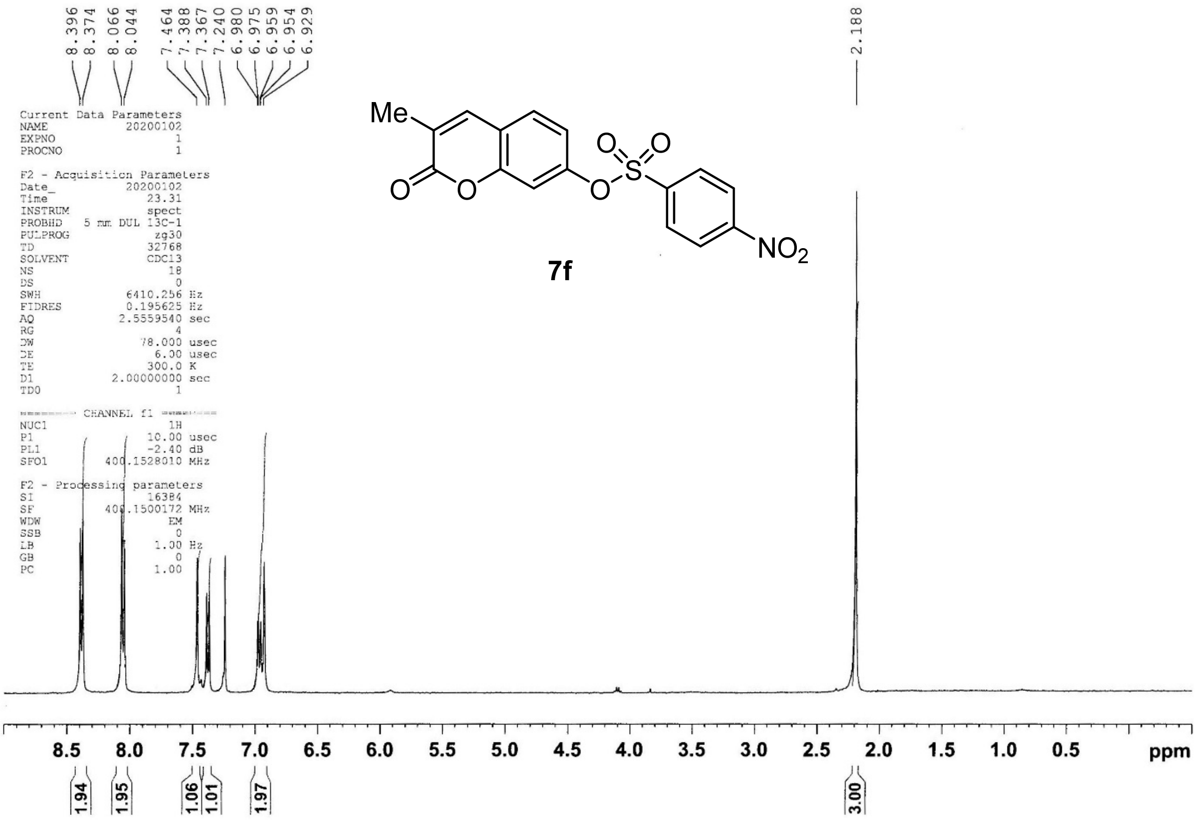

^{1}H NMR spectrum of compound **7f**

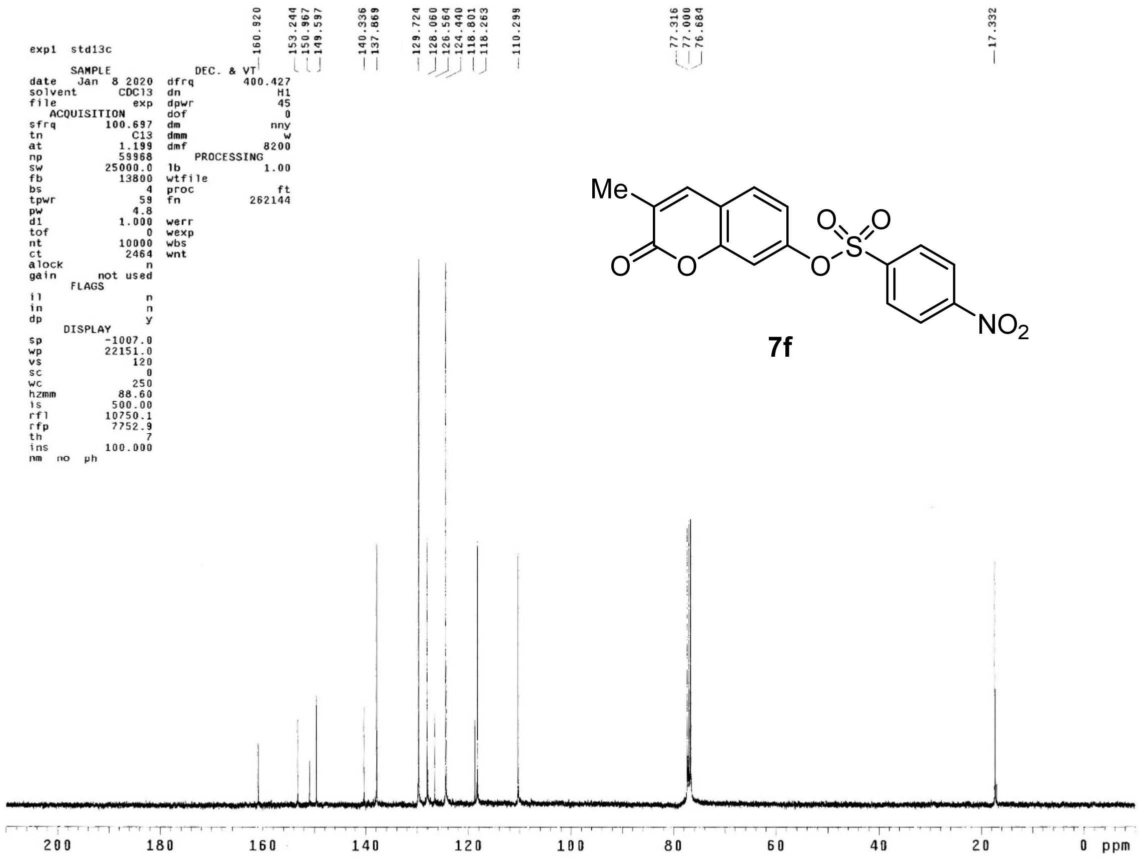

^{13}C NMR spectrum of compound **7f**

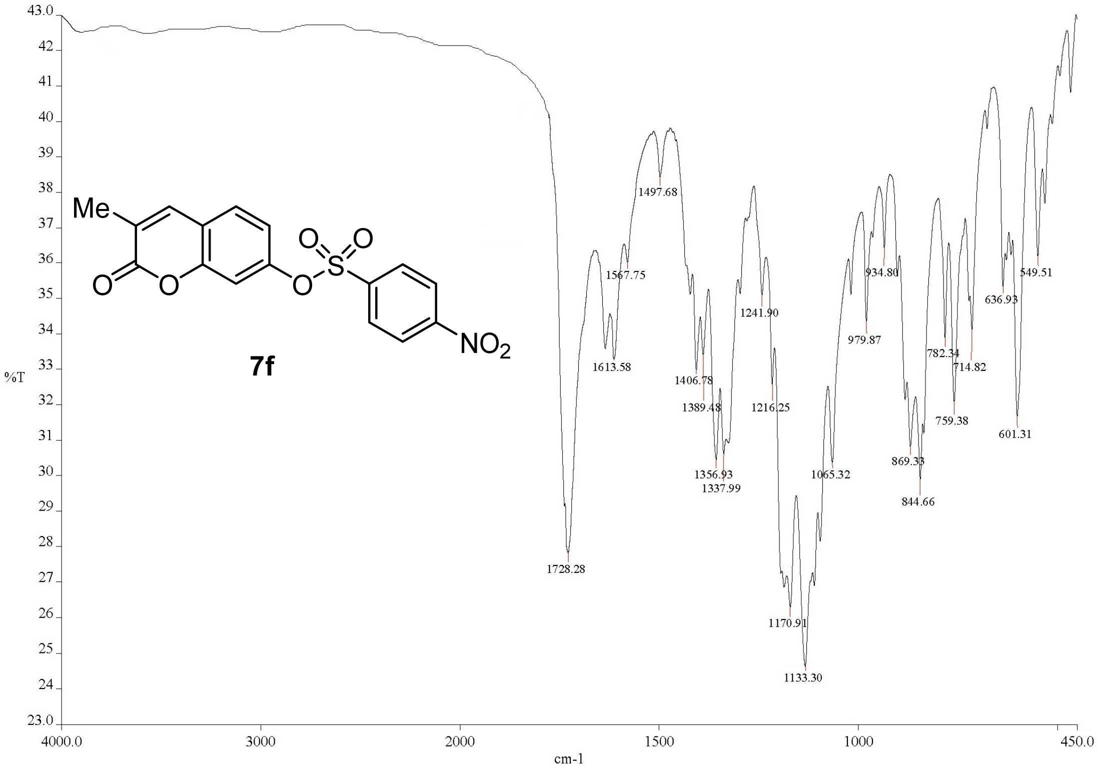

IR spectrum of compound **7f**

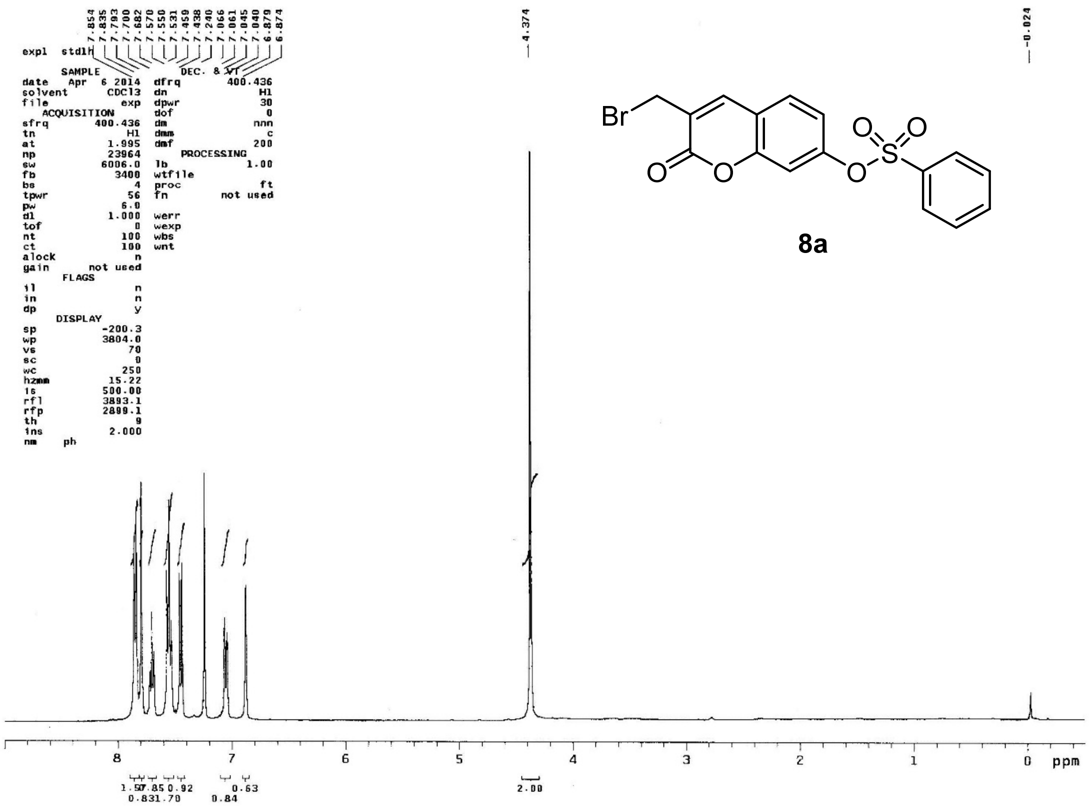

^{1}H NMR spectrum of compound **8a**

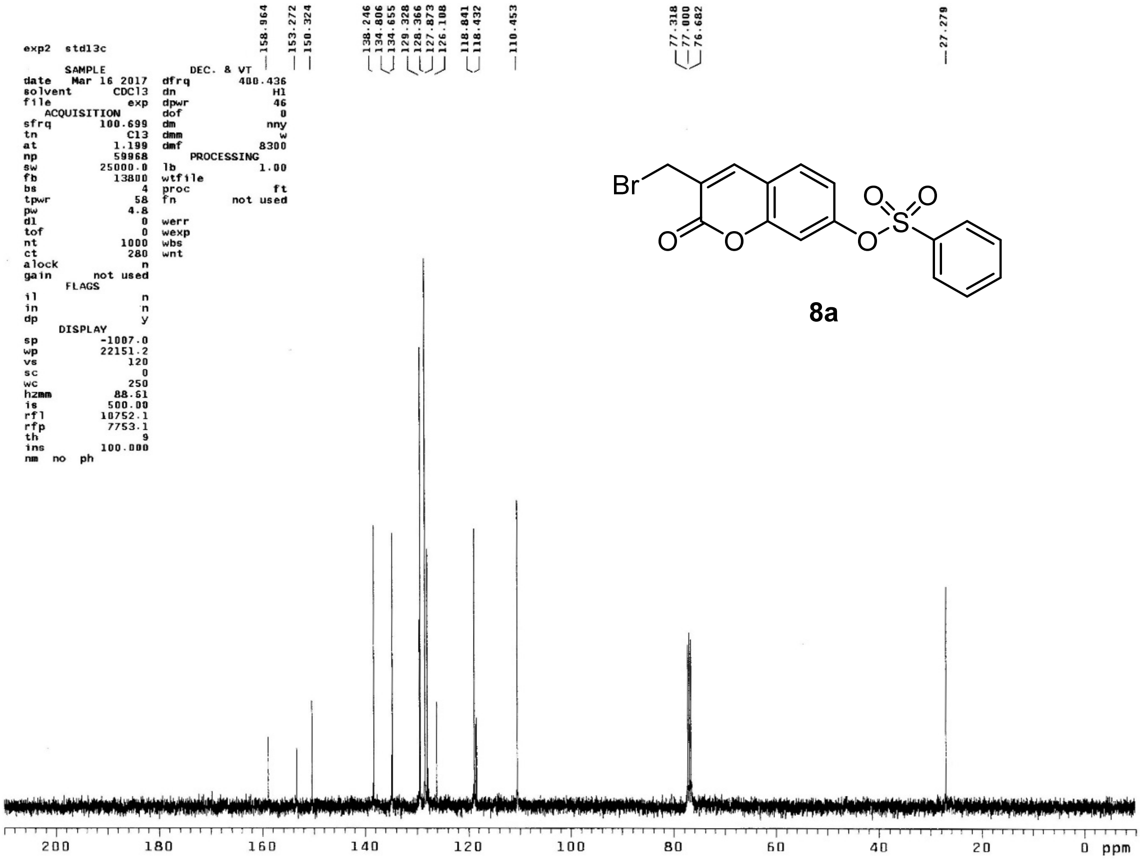

^{13}C NMR spectrum of compound **8a**

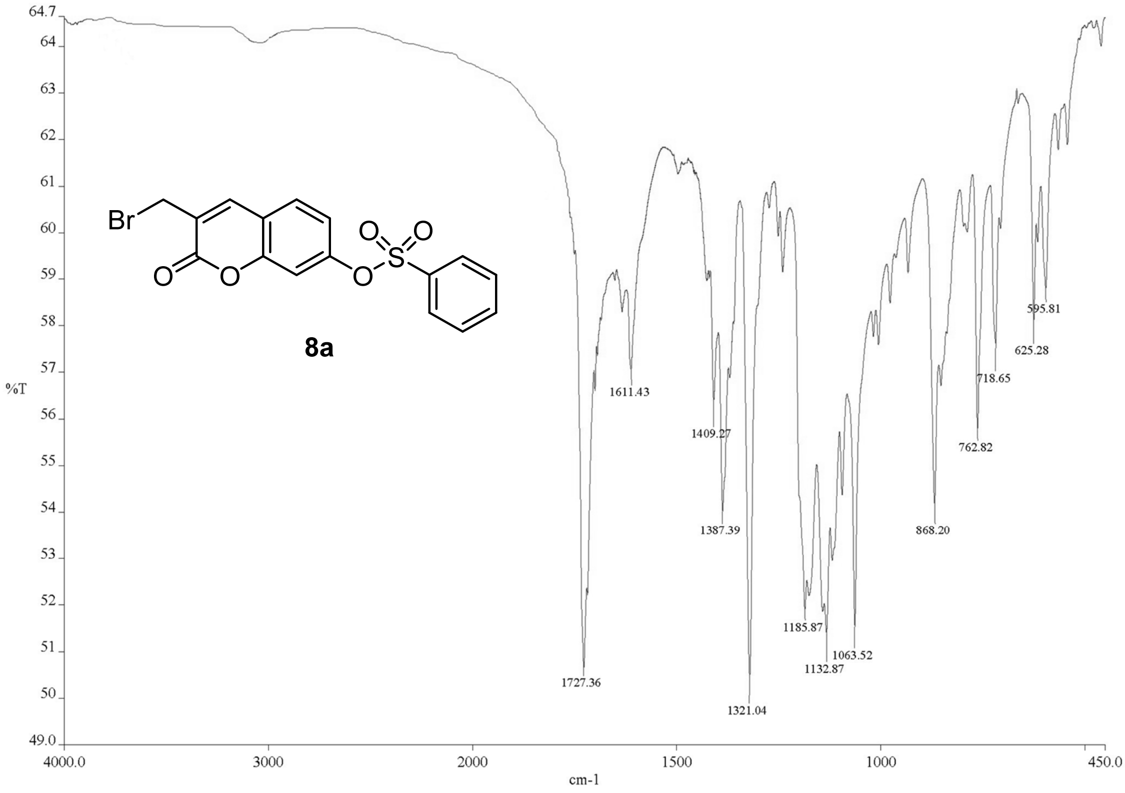

IR spectrum of compound **8a**

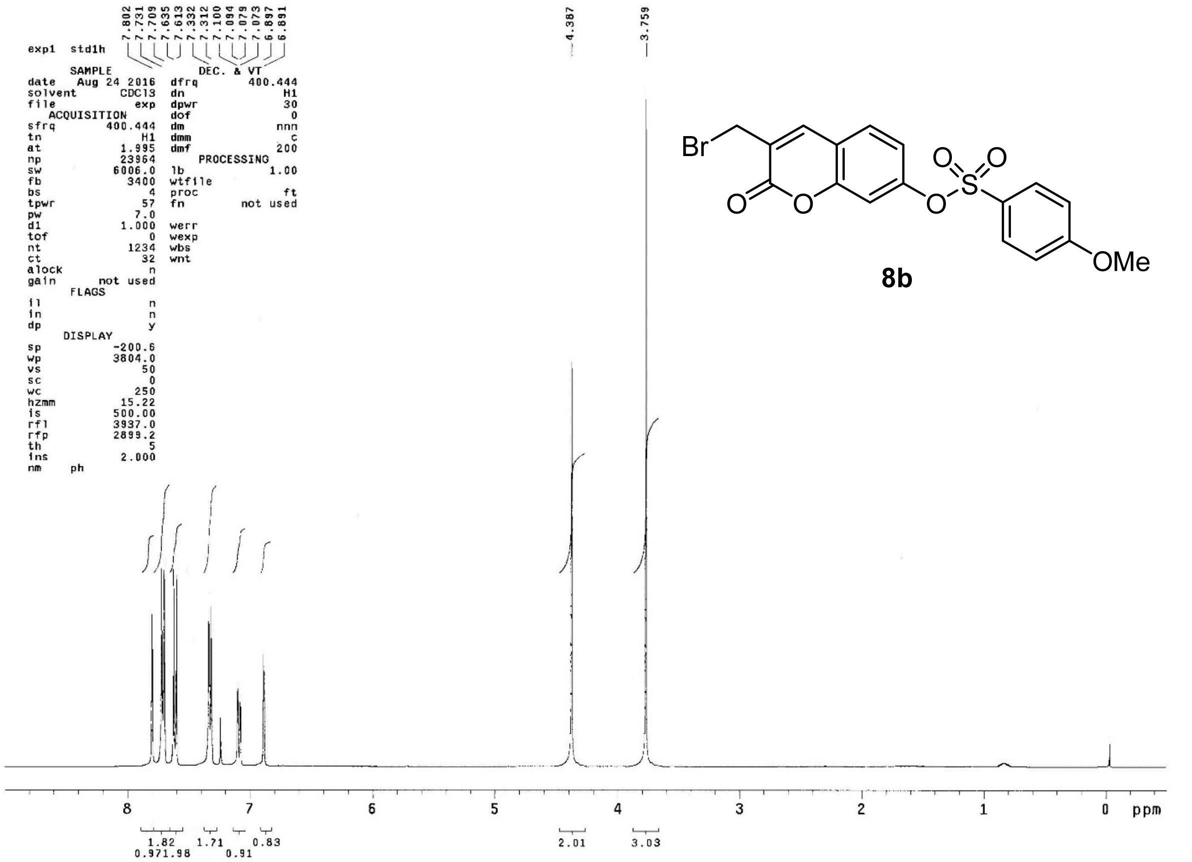

^{1}H NMR spectrum of compound **8b**

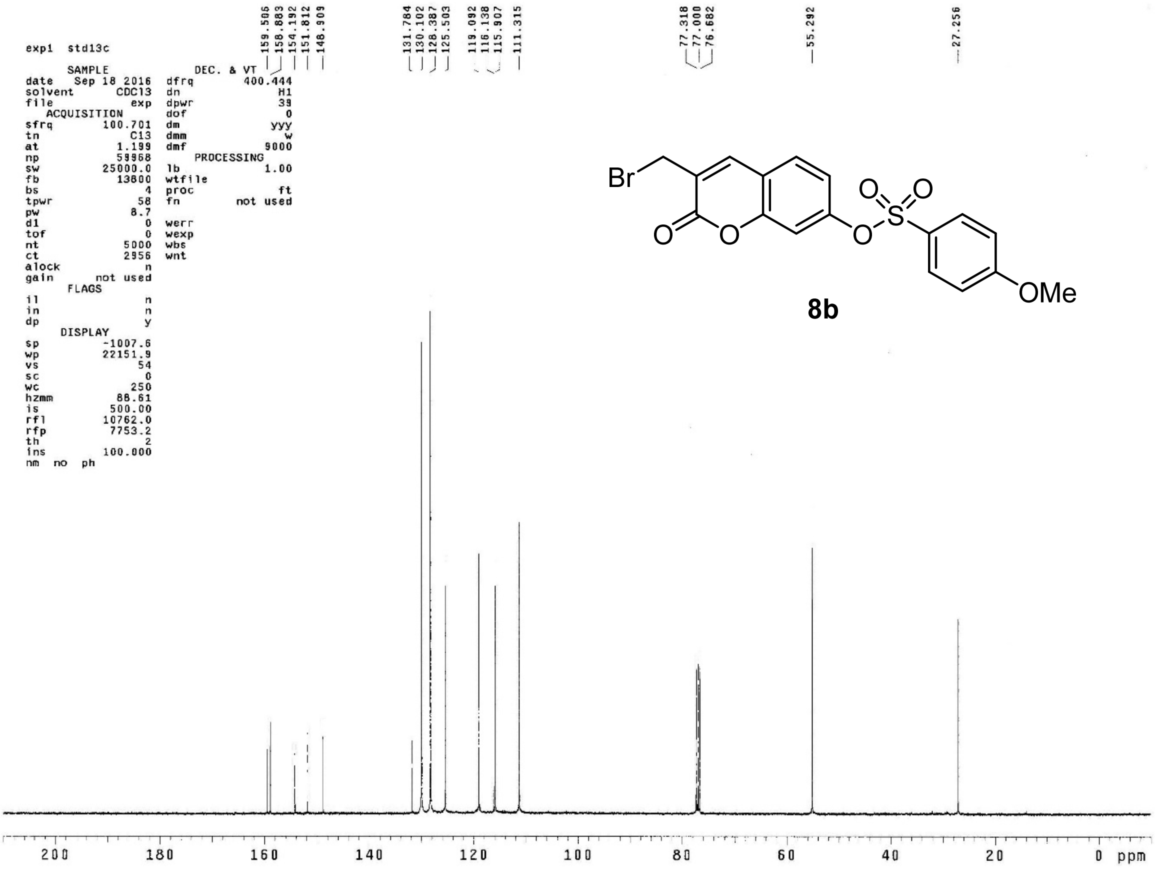

^{13}C NMR spectrum of compound **8b**

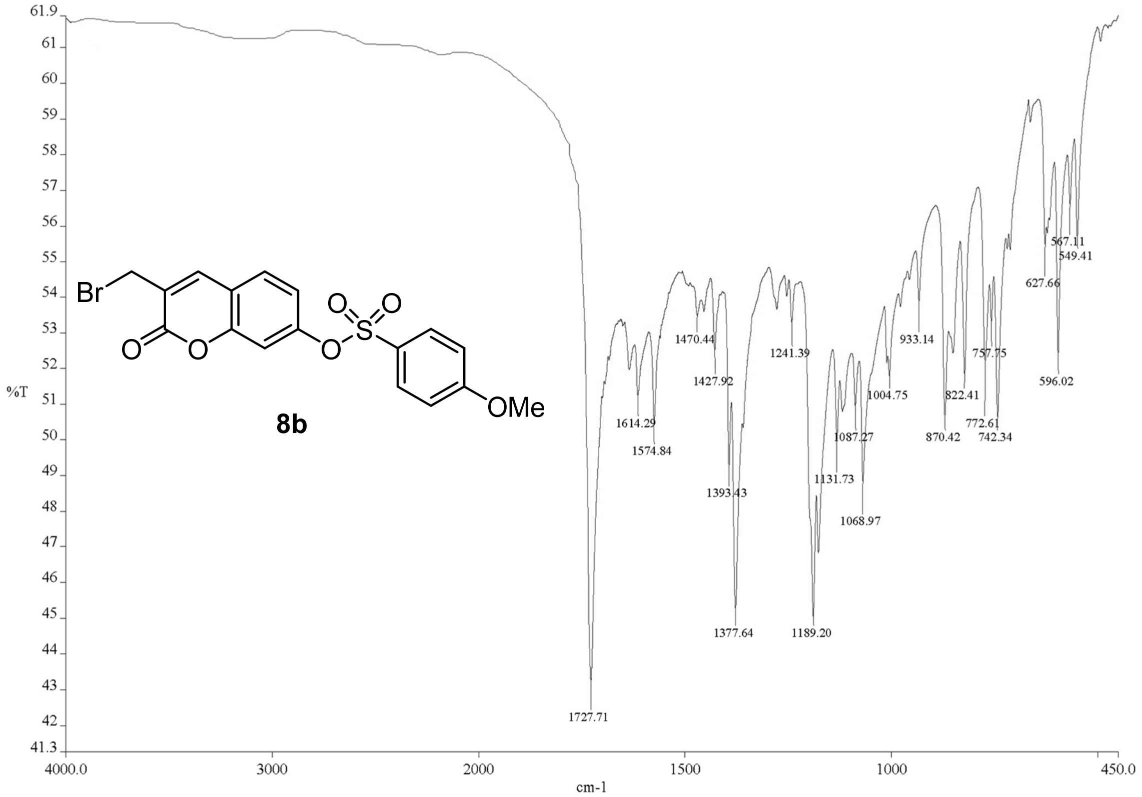

IR spectrum of compound **8b**

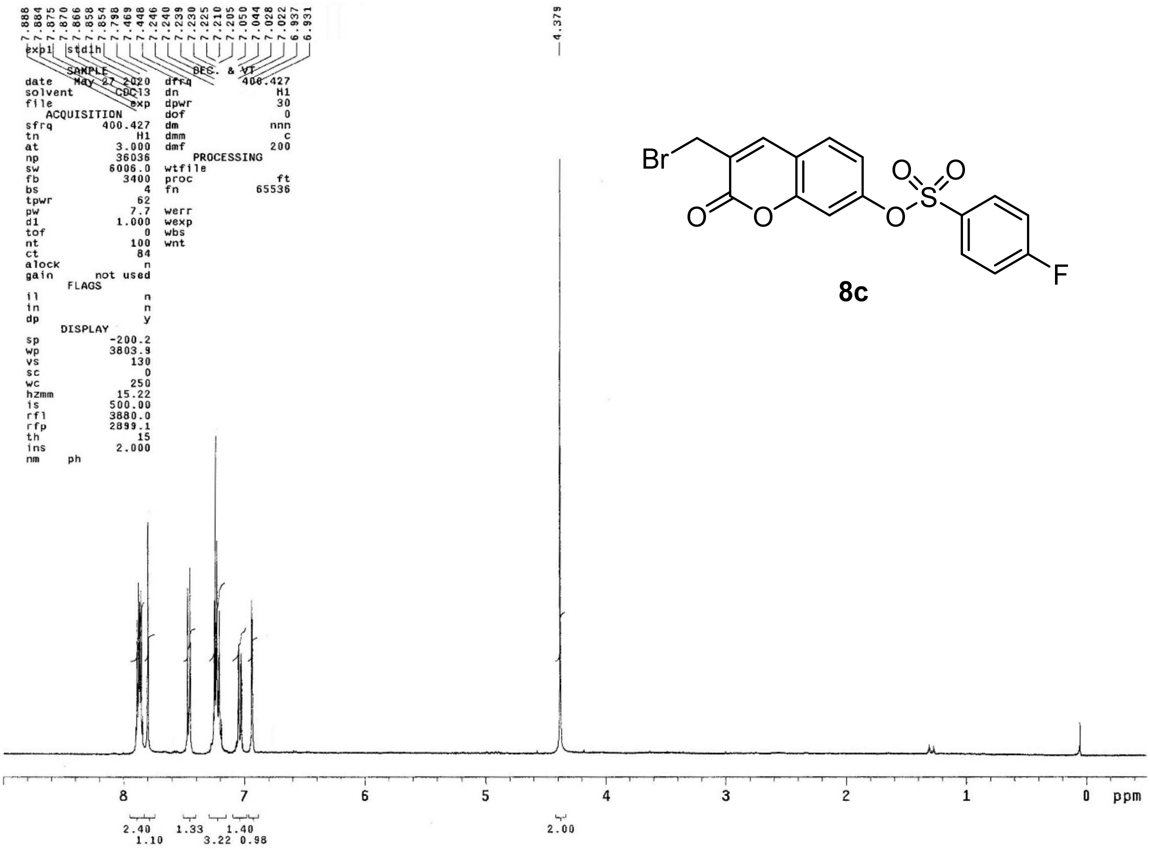

^{1}H NMR spectrum of compound **8c**

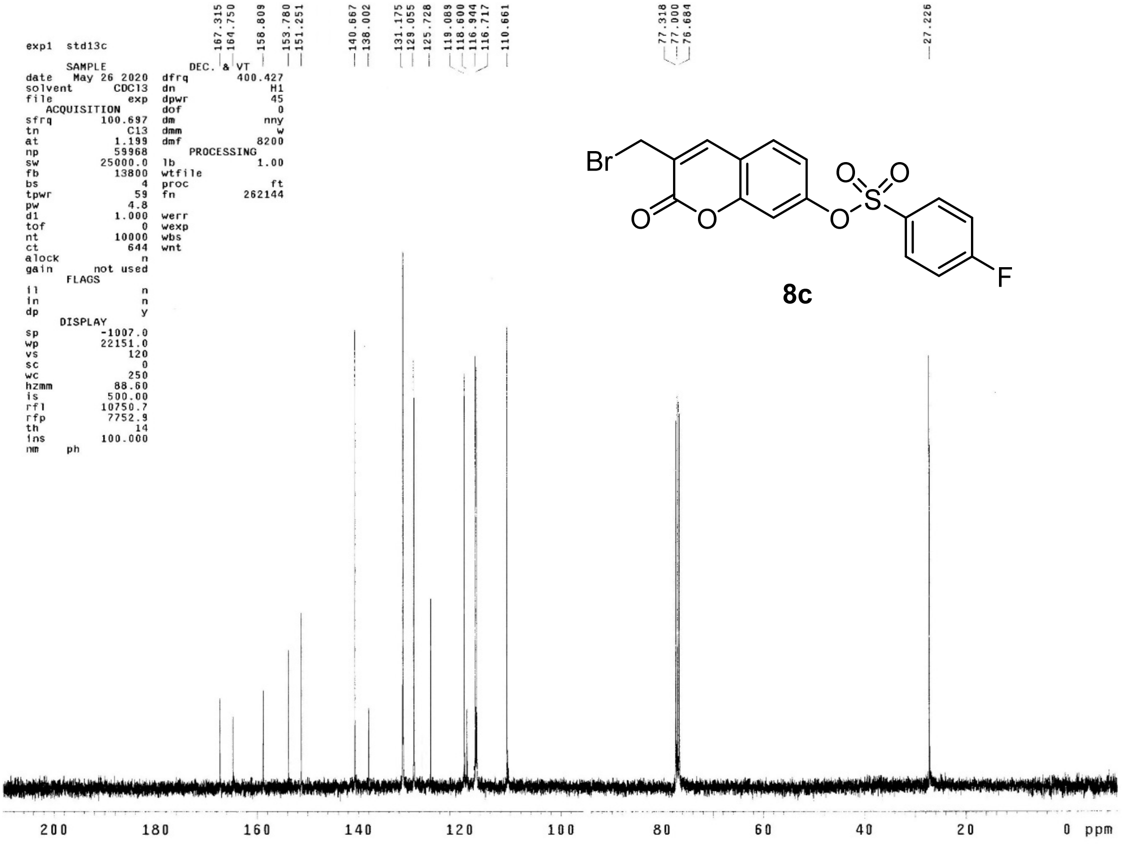

^{13}C NMR spectrum of compound **8c**

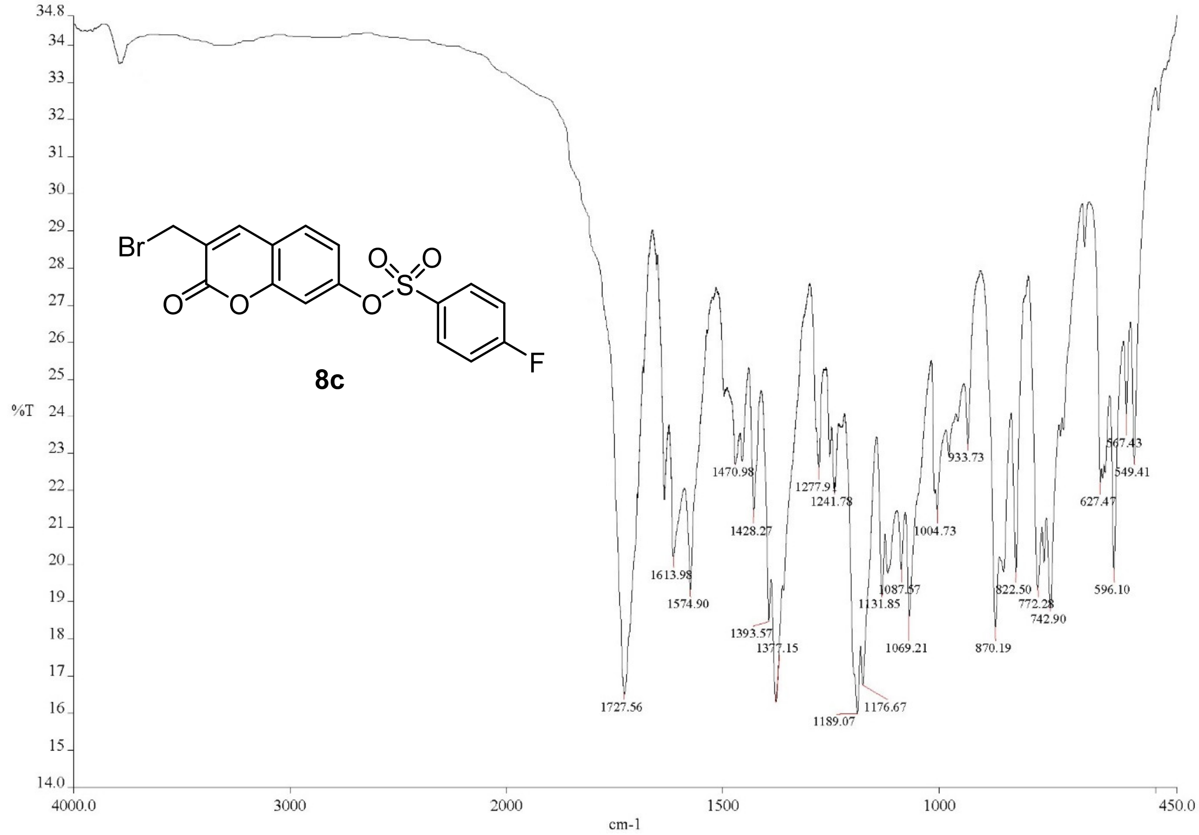

IR spectrum of compound **8c**

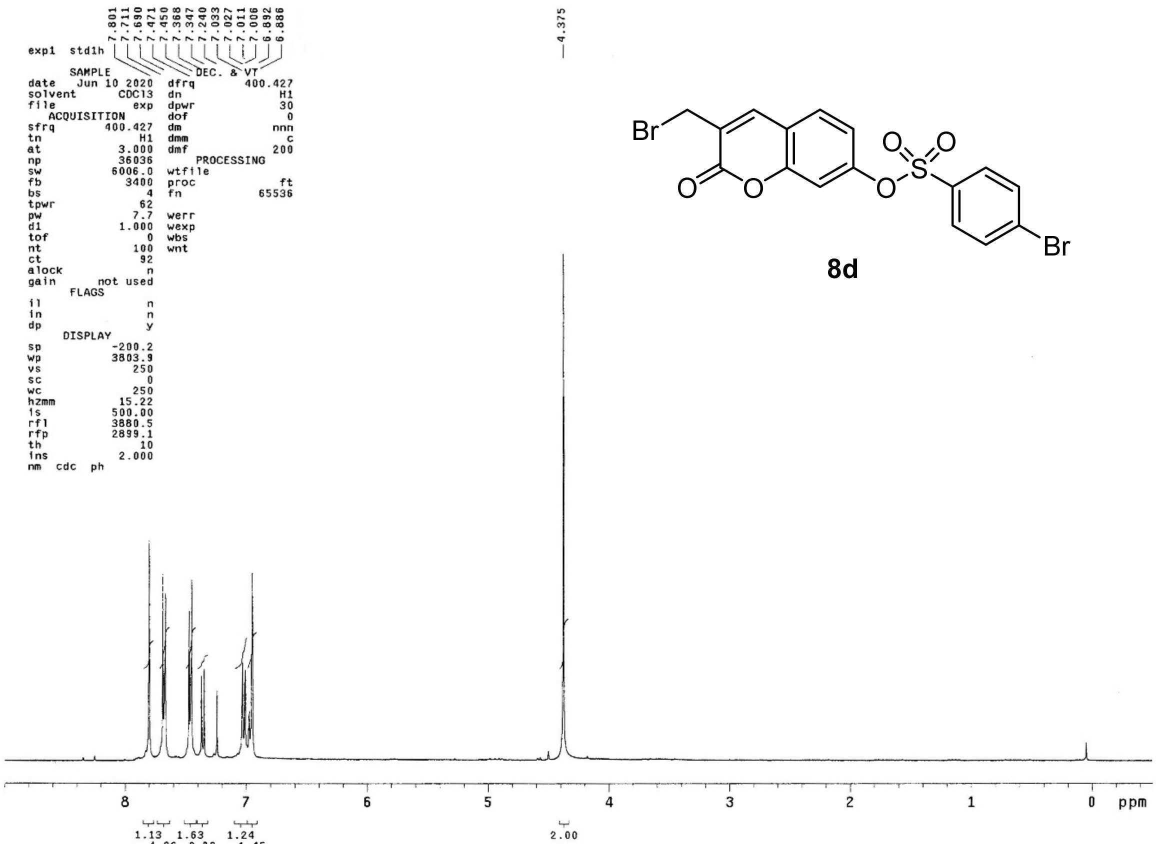

^{1}H NMR spectrum of compound **8d**

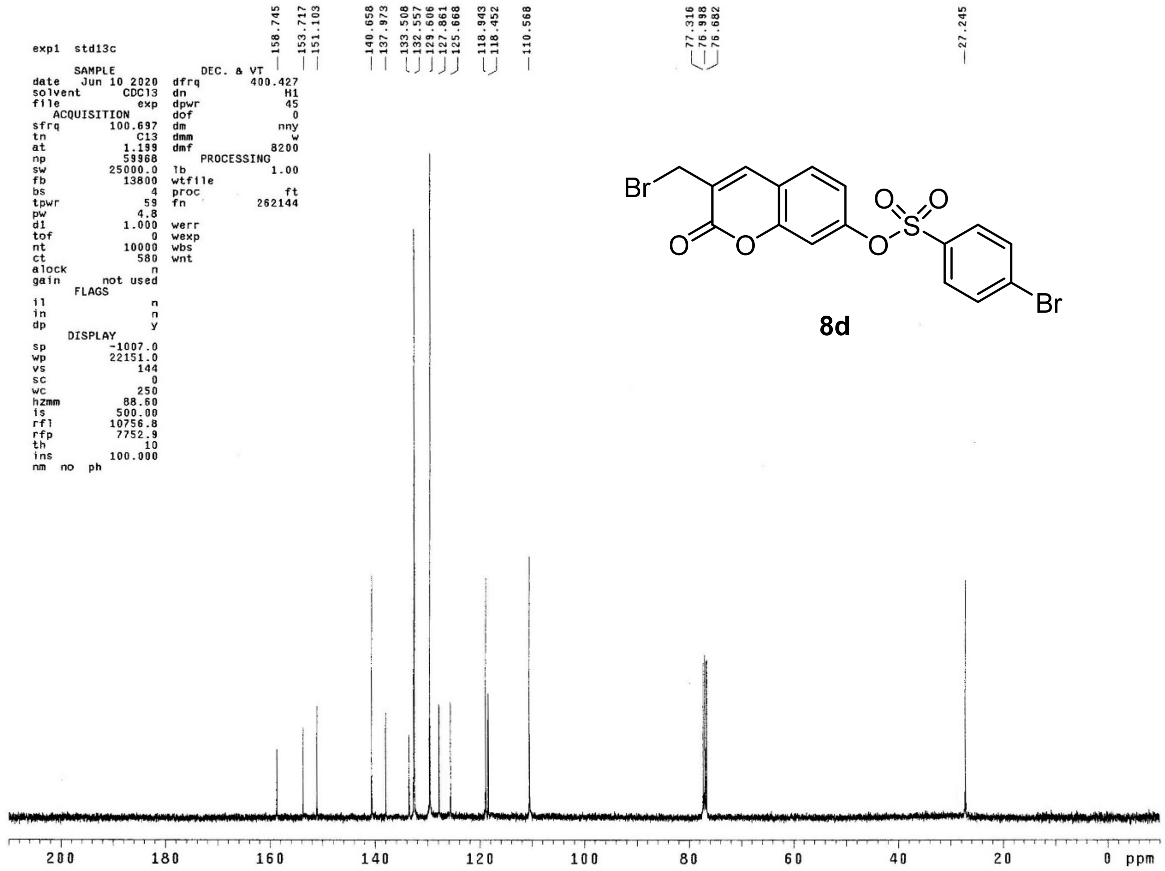

^{13}C NMR spectrum of compound **8d**

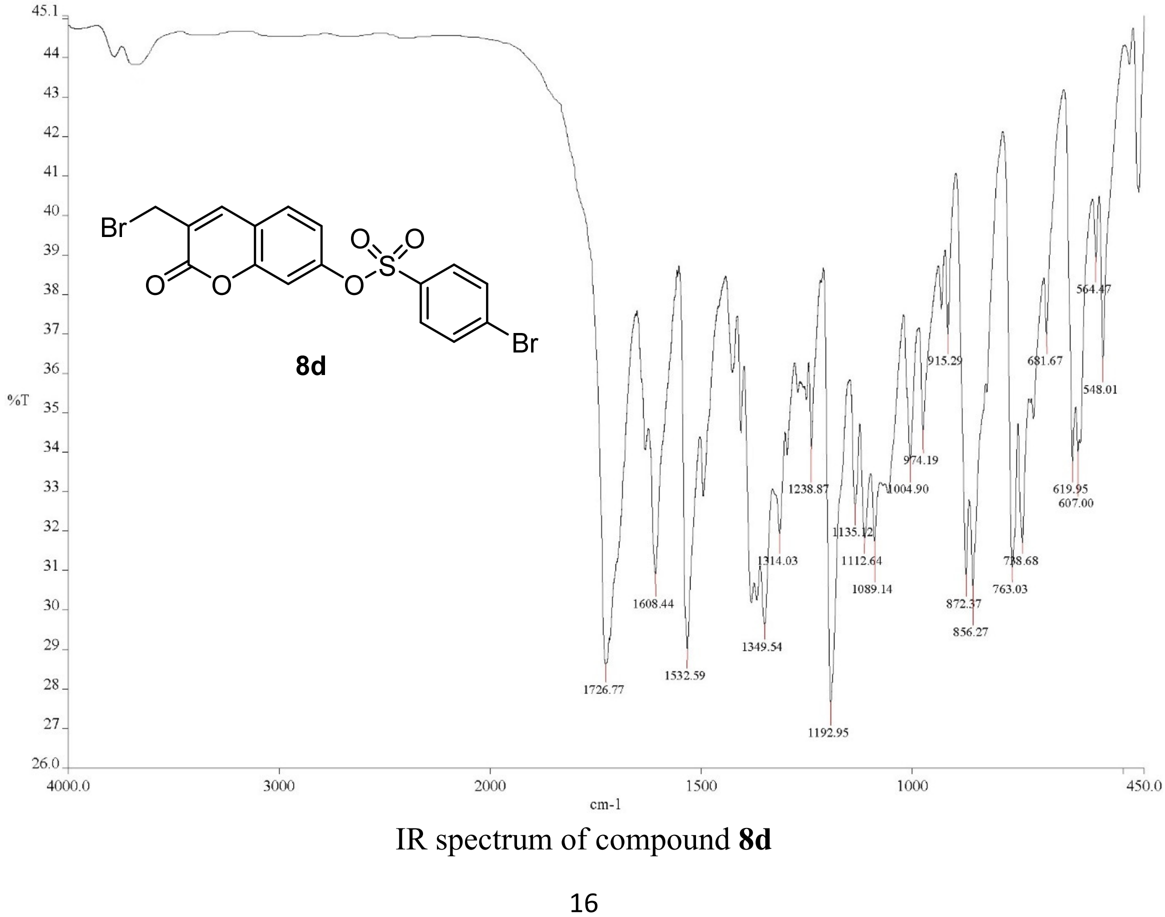

IR spectrum of compound **8d**

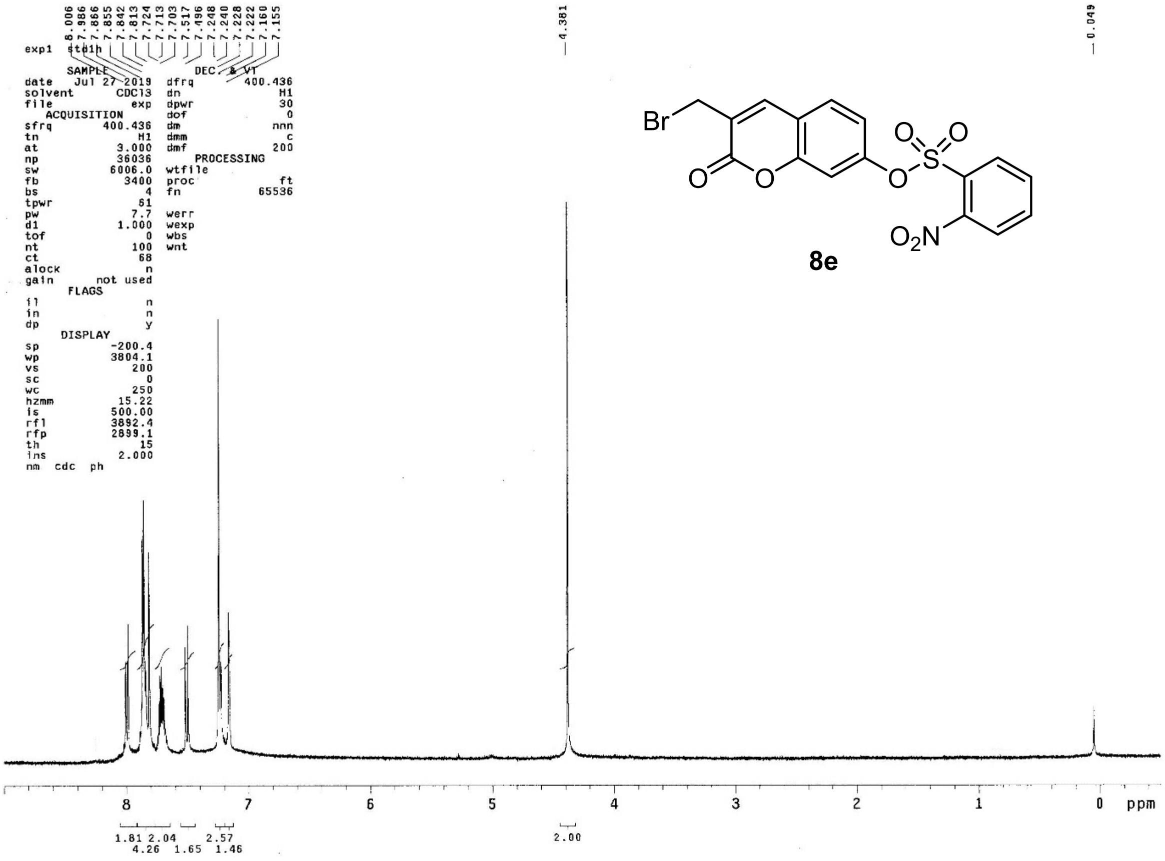

^{1}H NMR spectrum of compound **8e**

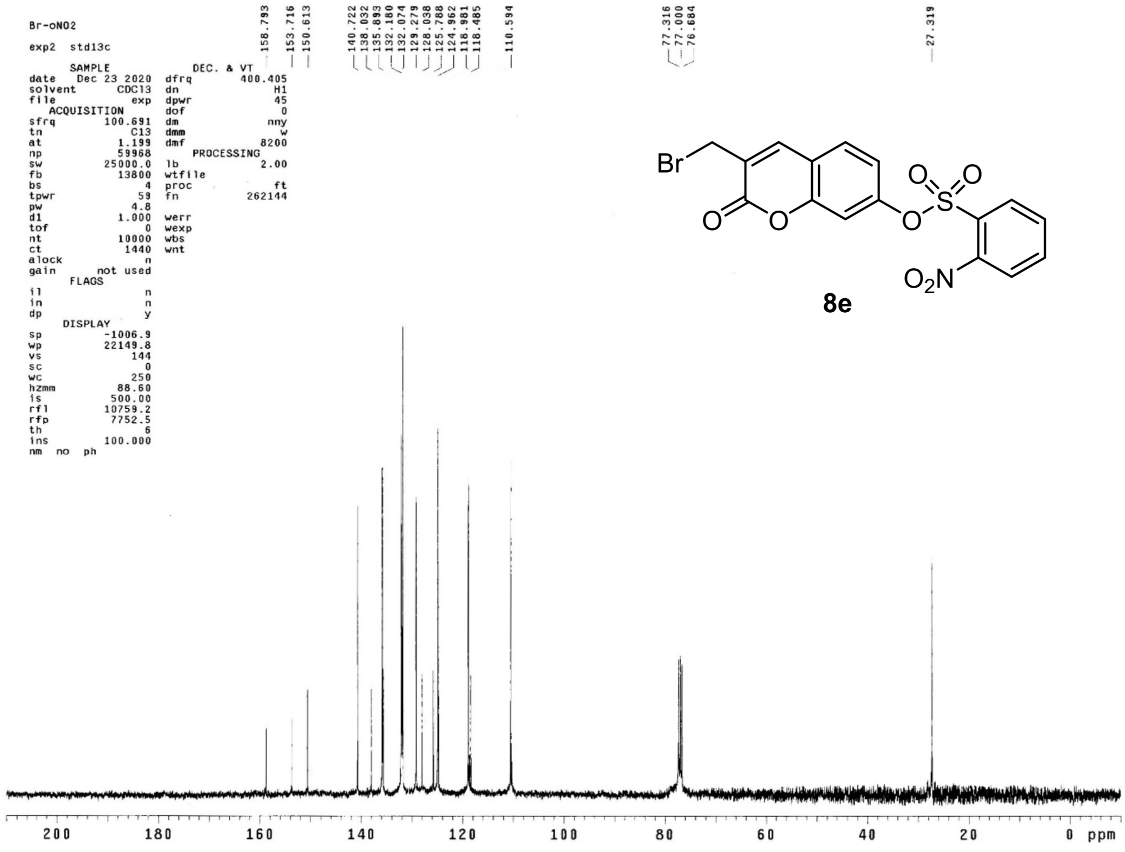

^{13}C NMR spectrum of compound **8e**

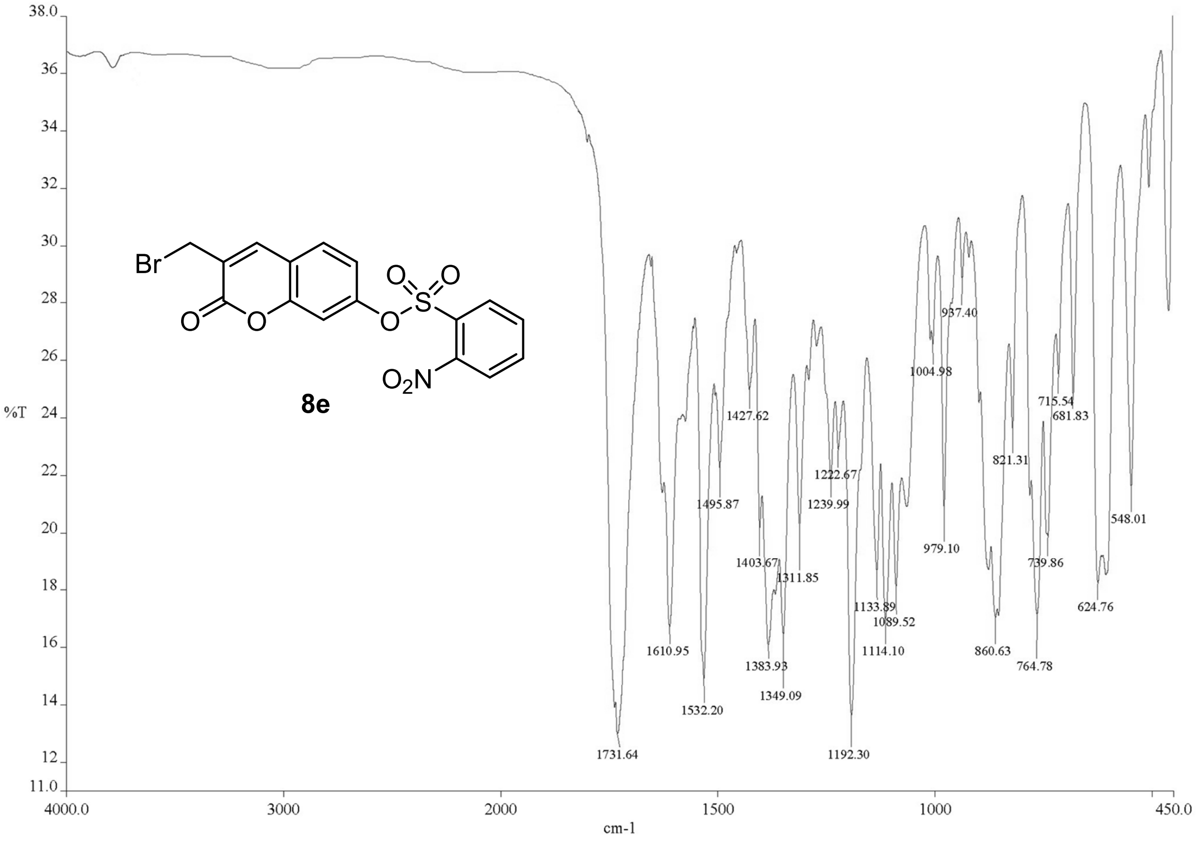

IR spectrum of compound **8e**

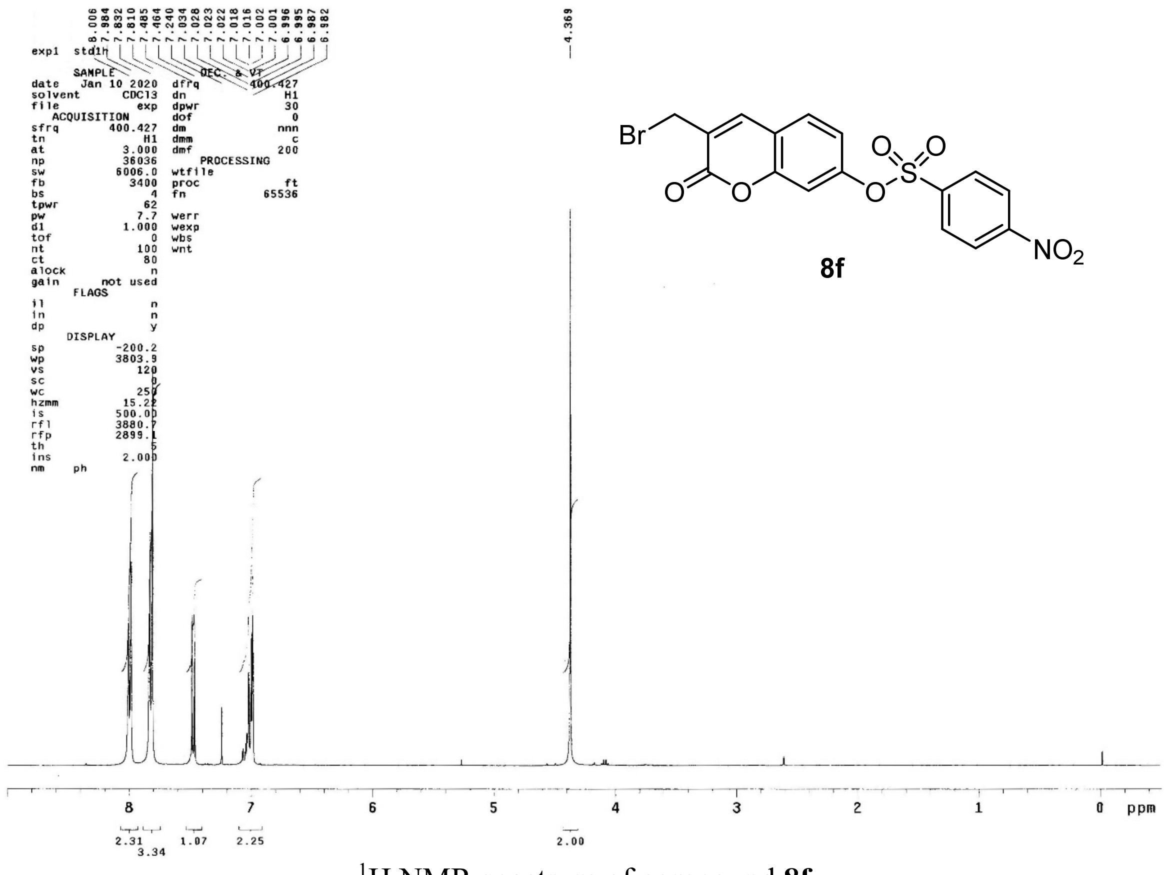

^{1}H NMR spectrum of compound **8f**

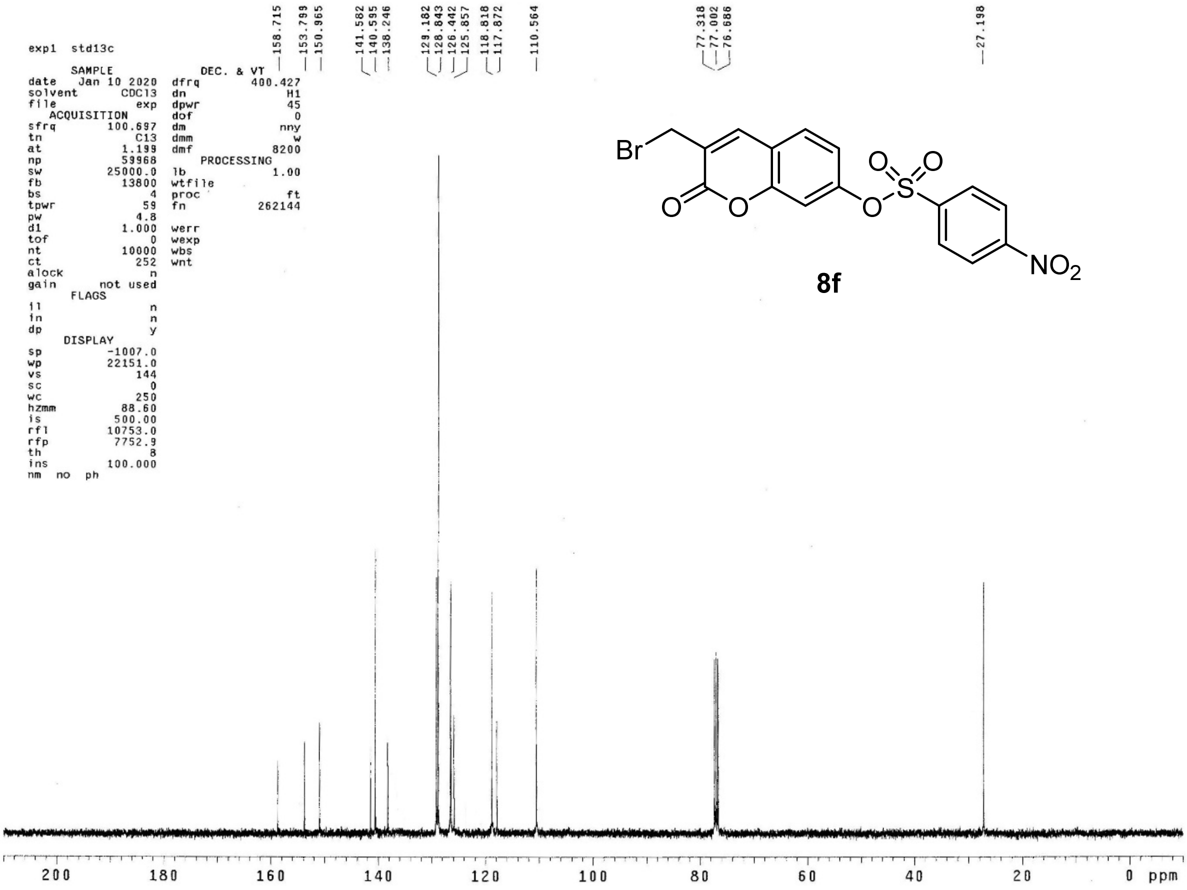

^{13}C NMR spectrum of compound **8f**

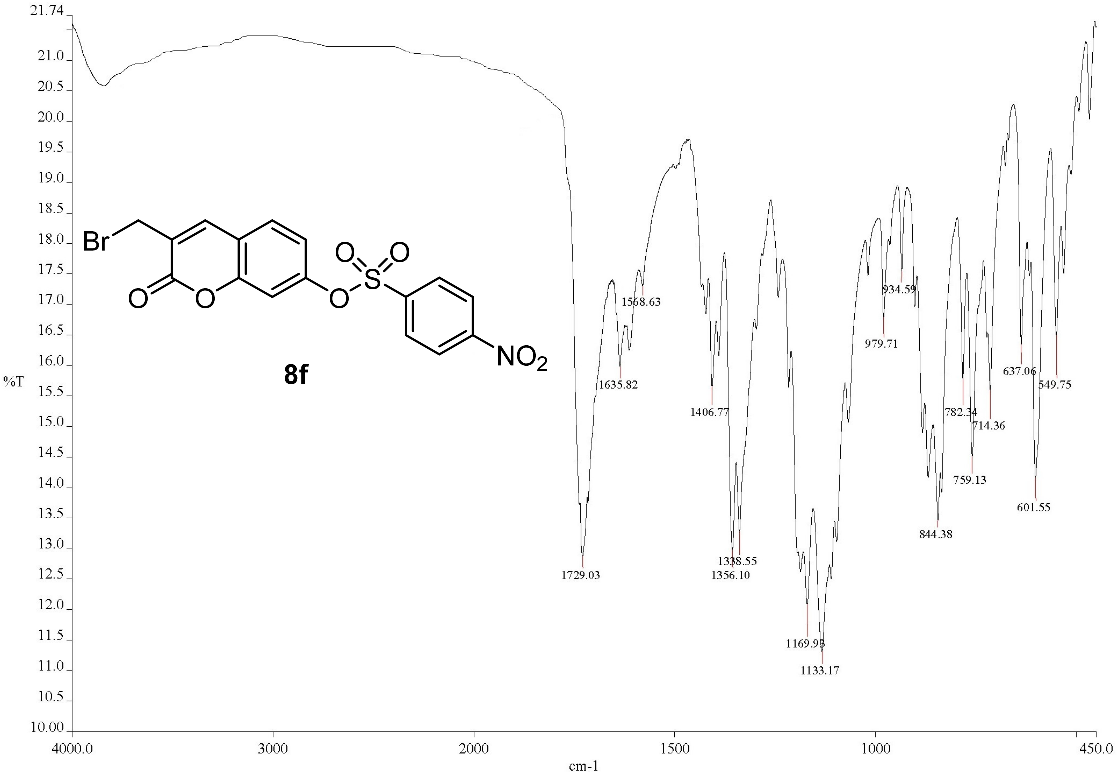

IR spectrum of compound **8f**

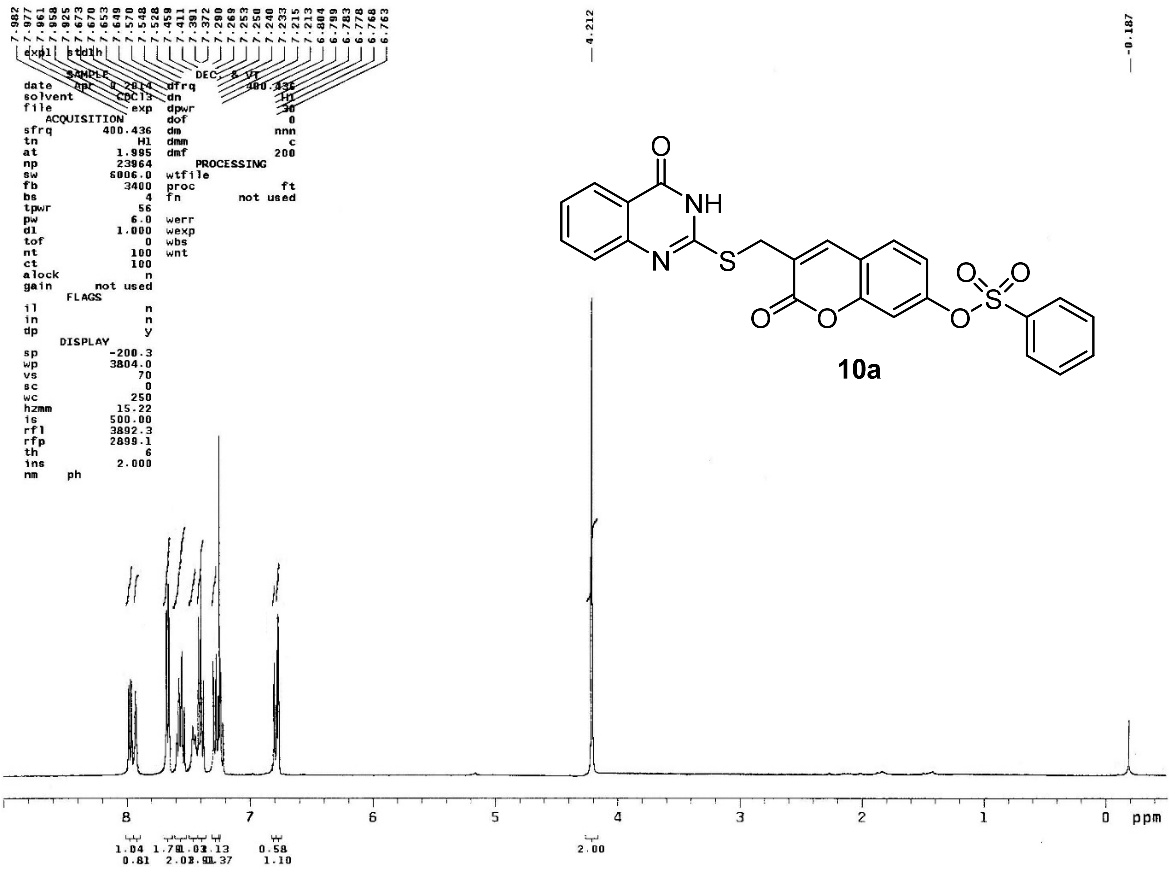

^{1}H NMR spectrum of compound **10a**

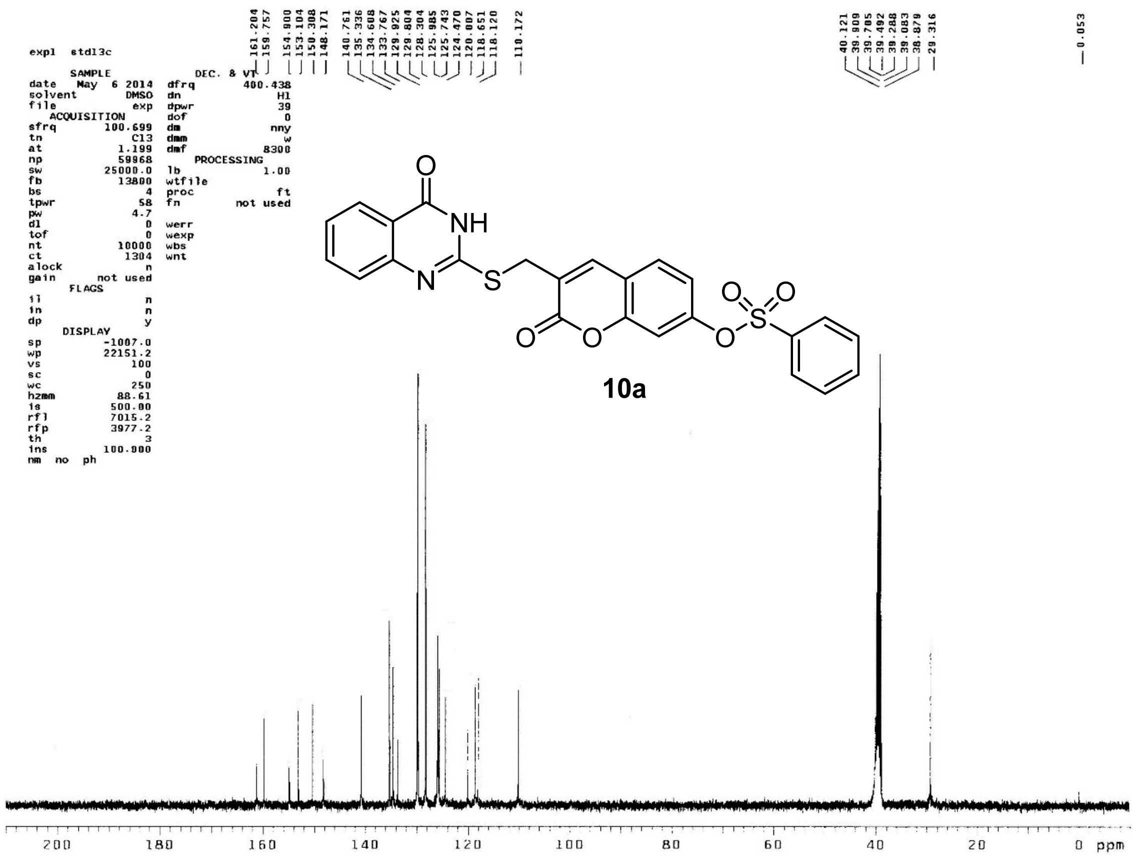

^{13}C NMR spectrum of compound **10a**

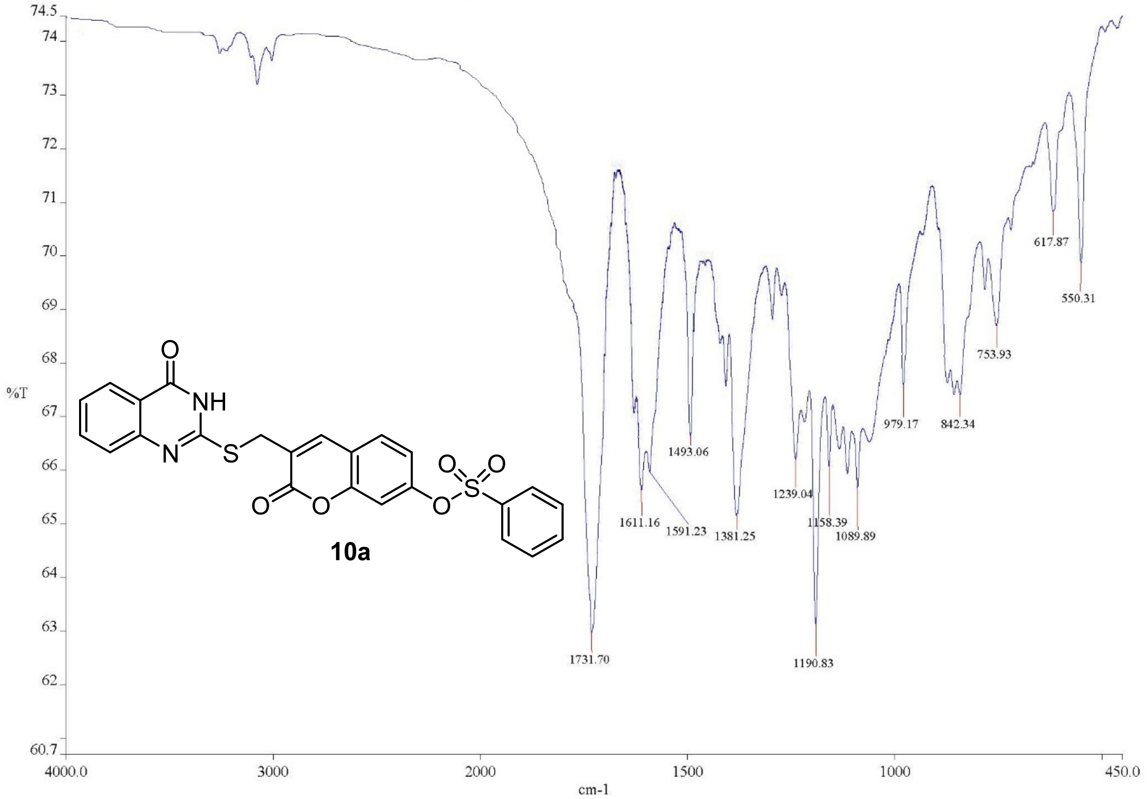

IR spectrum of compound **10a**

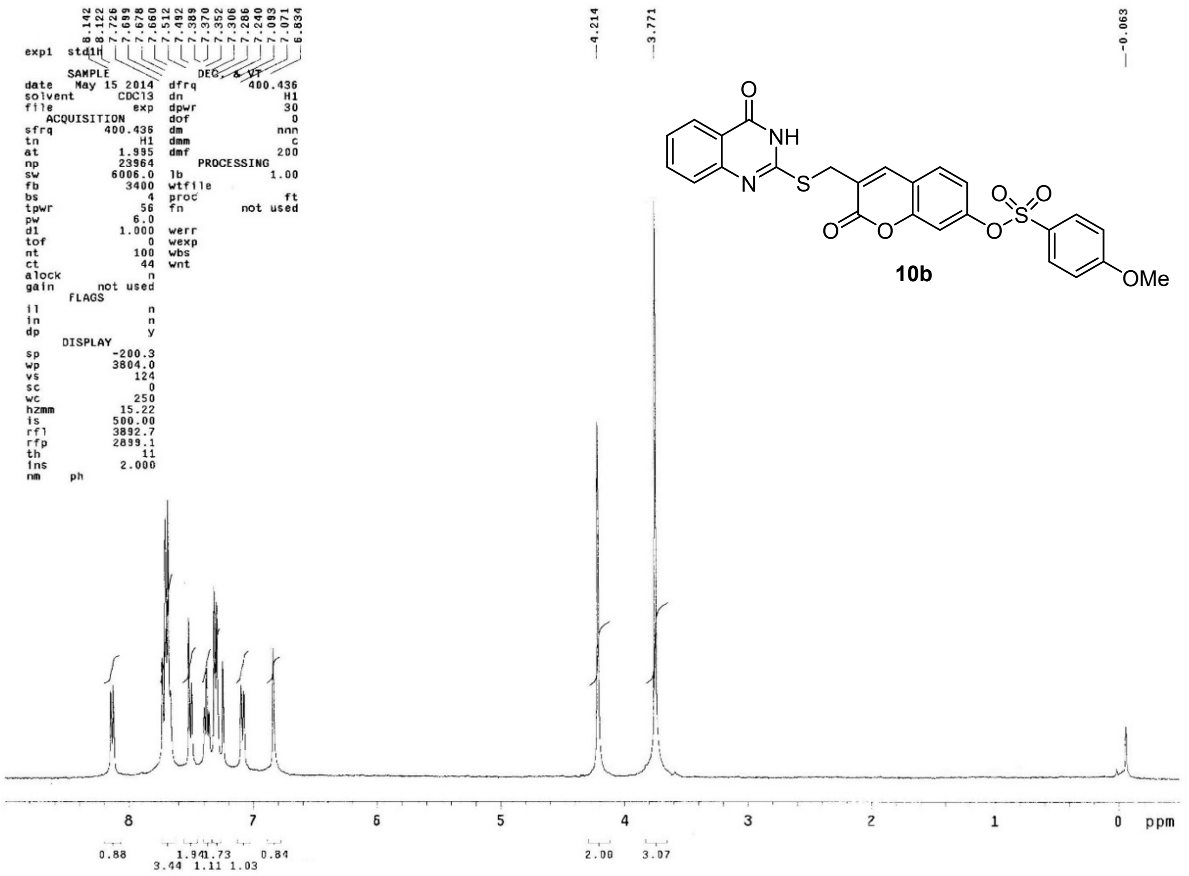

^{1}H NMR spectrum of compound **10b**

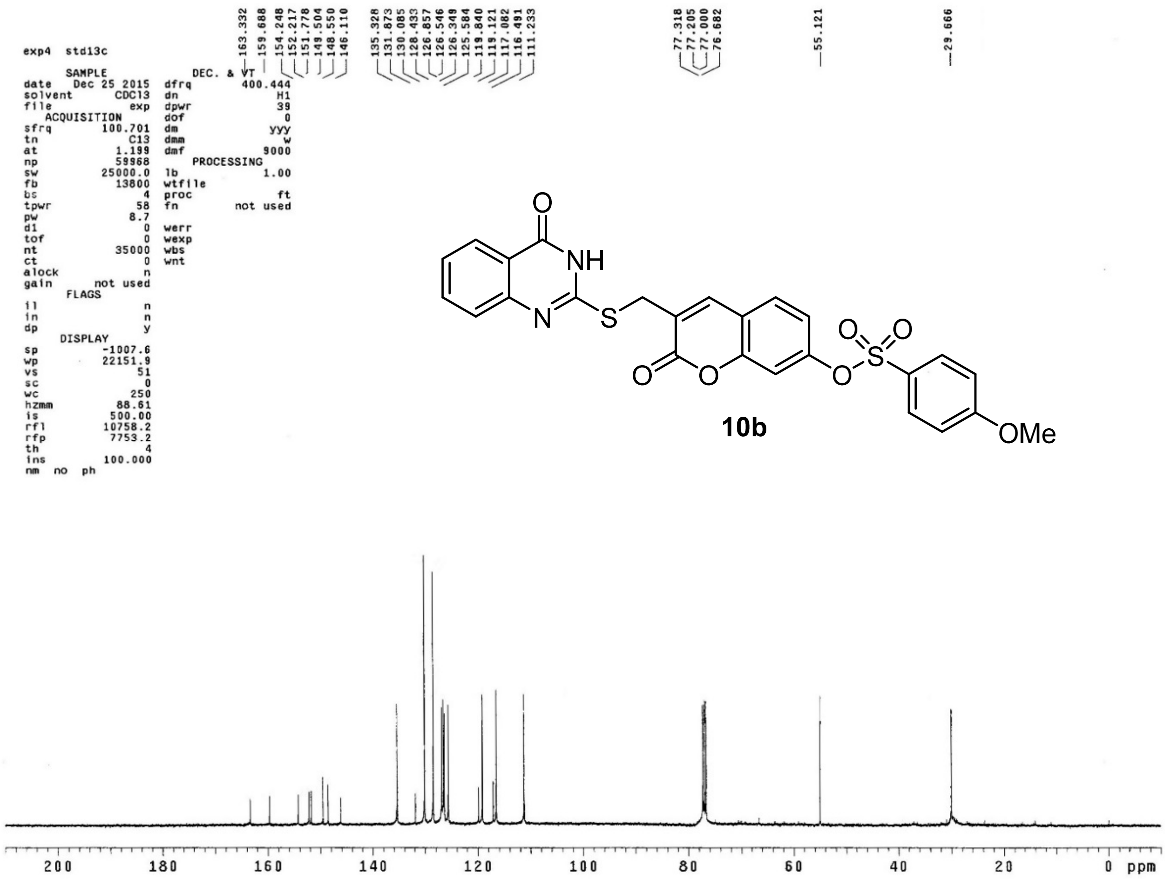

^{13}C NMR spectrum of compound **10b**

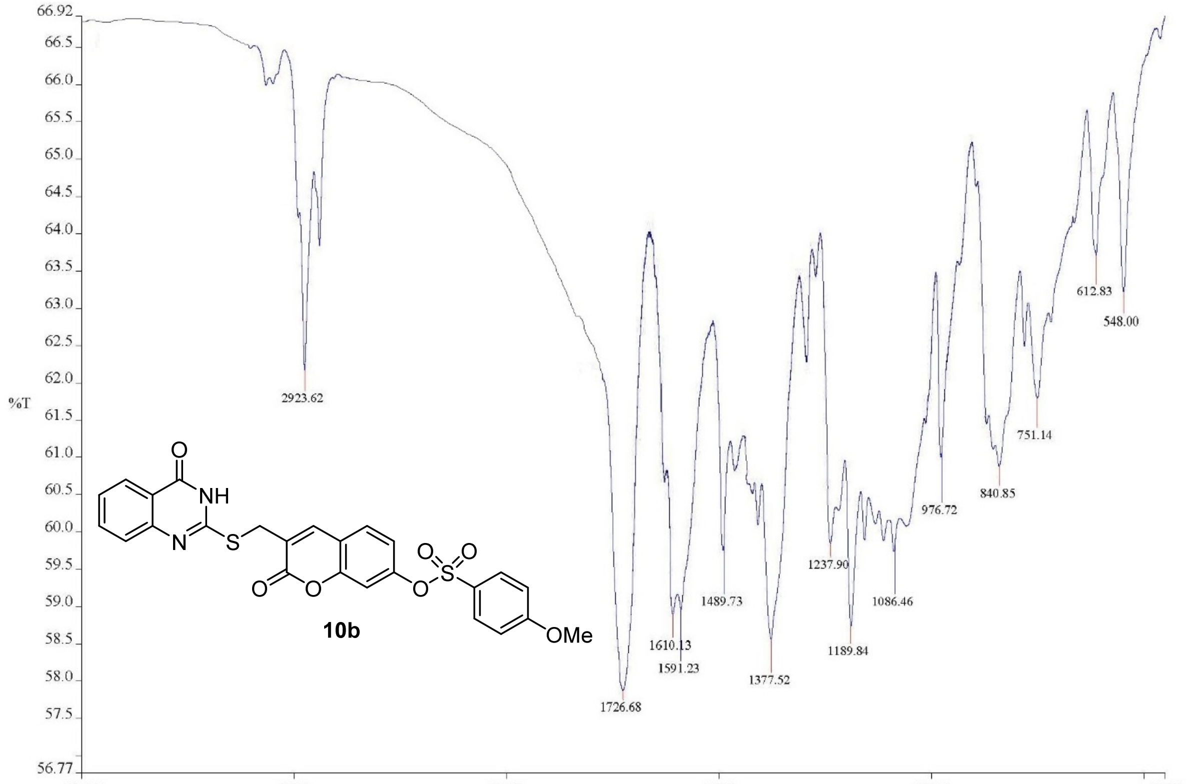

IR spectrum of compound **10b**

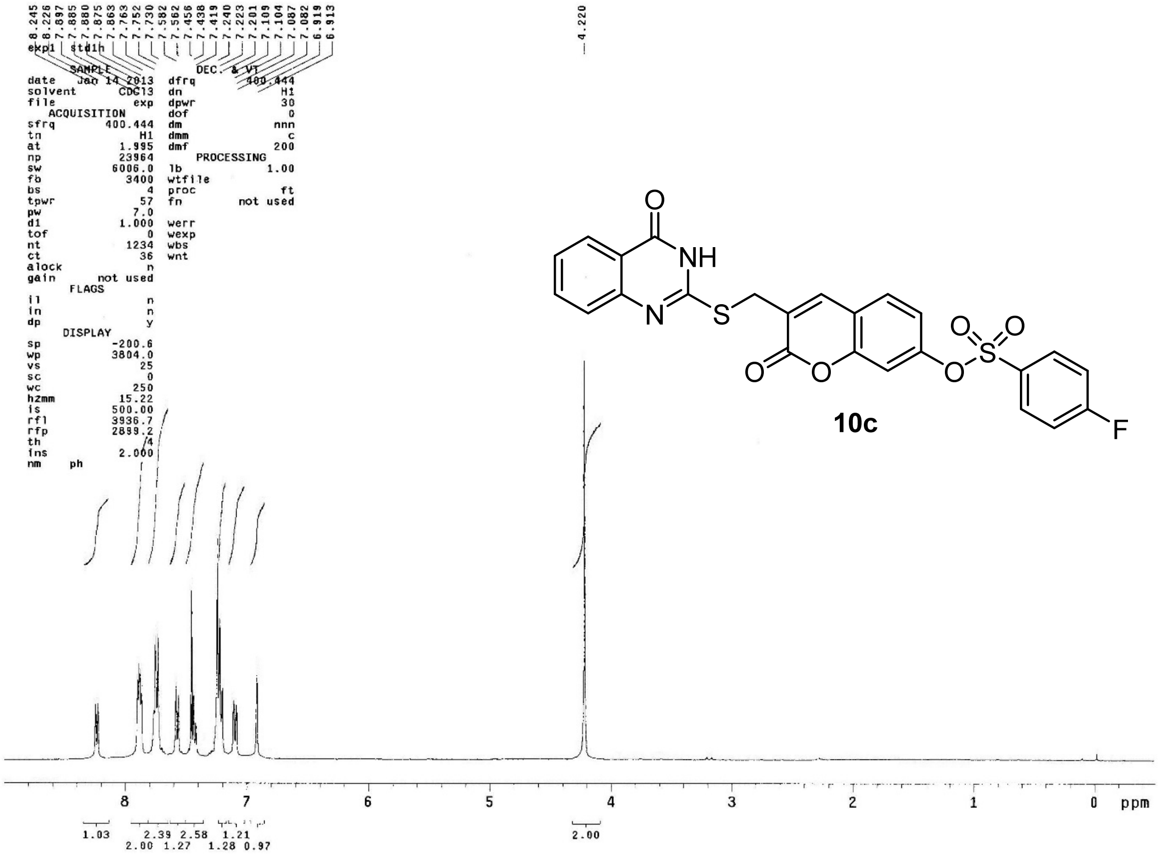

^{1}H NMR spectrum of compound **10c**

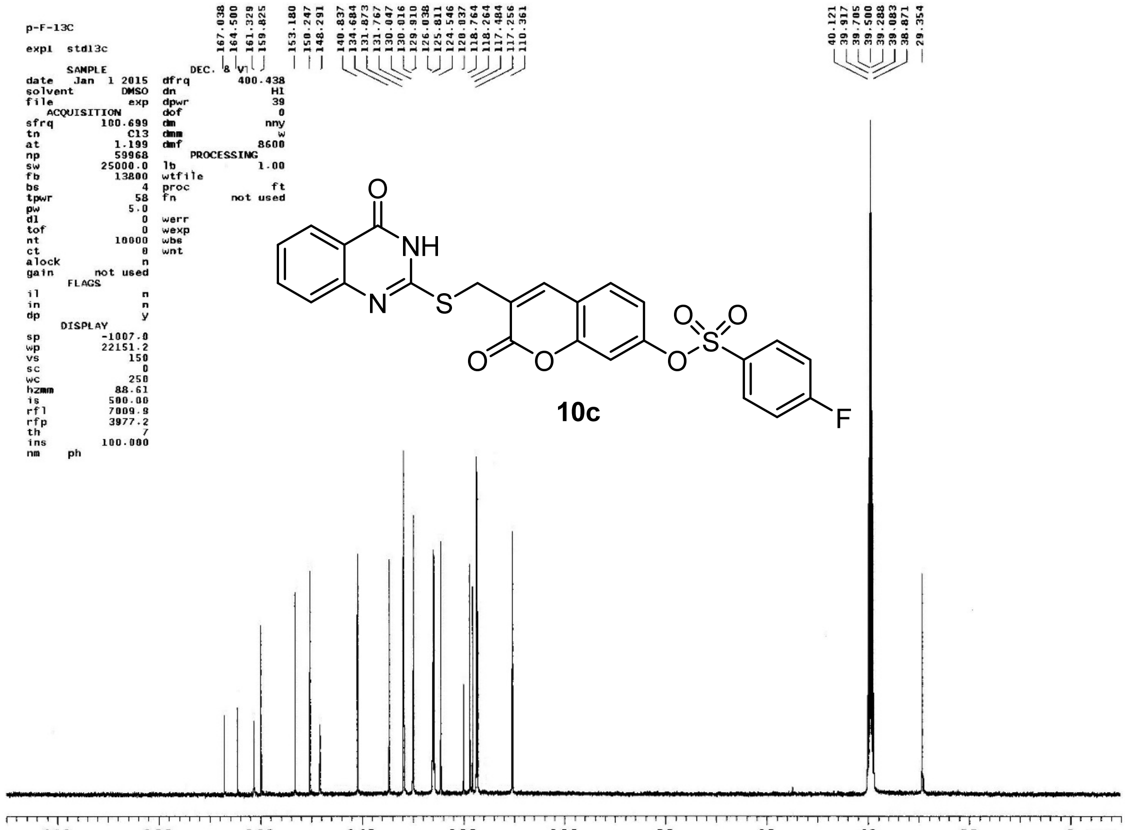

^{13}C NMR spectrum of compound **10c**

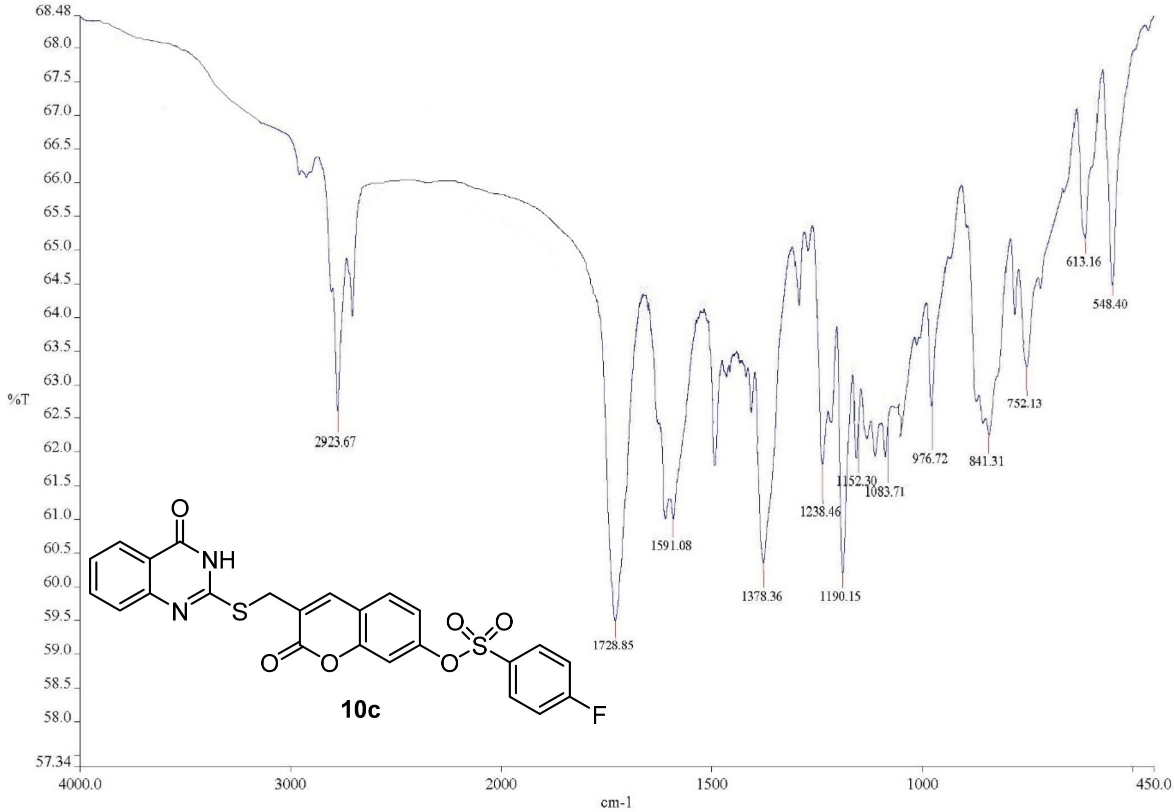

IR spectrum of compound **10c**

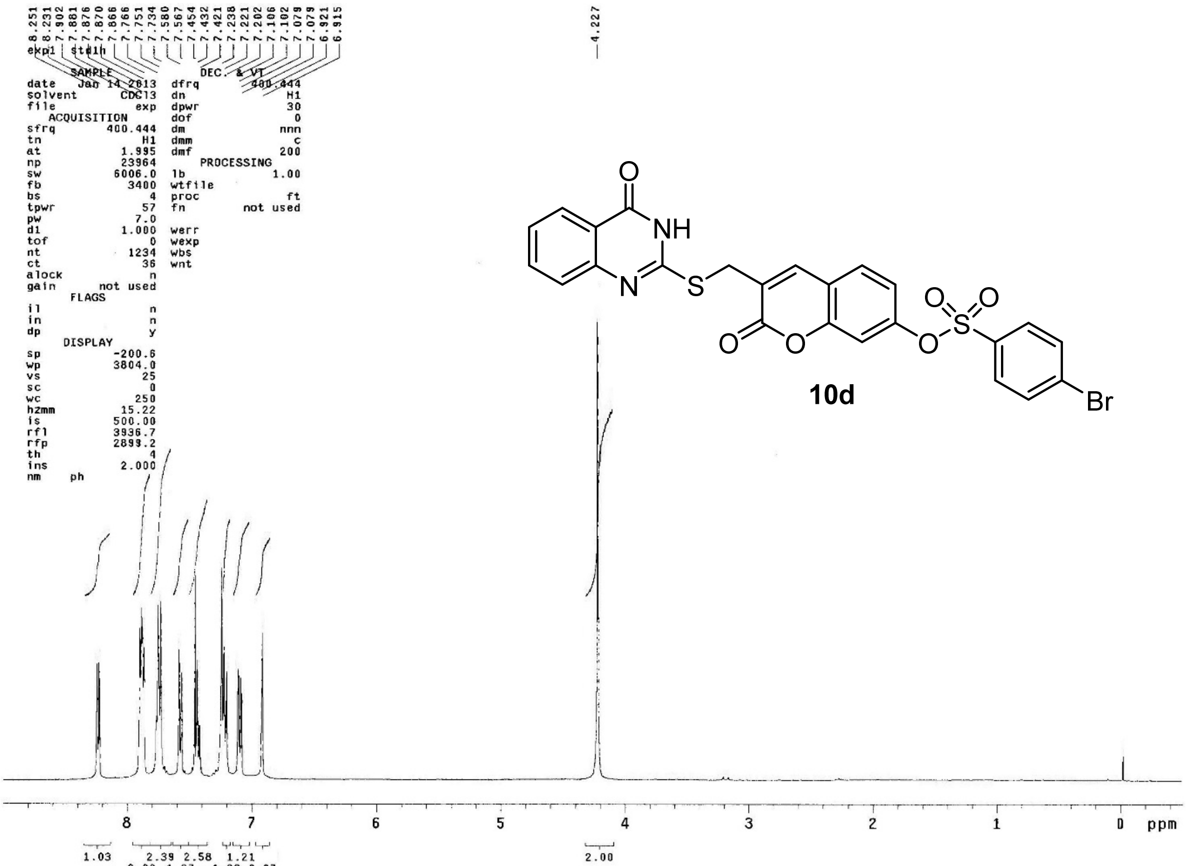

^{1}H NMR spectrum of compound **10d**

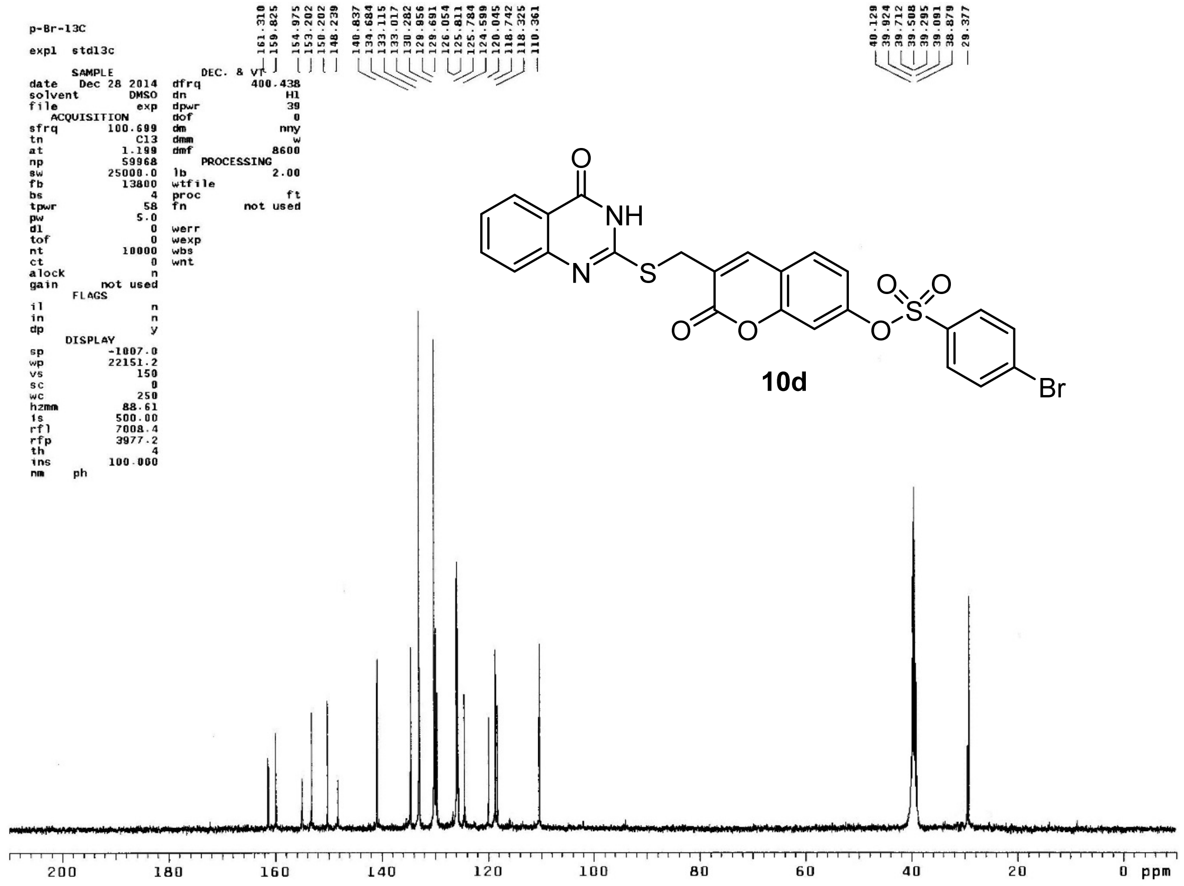

^{13}C NMR spectrum of compound **10d**

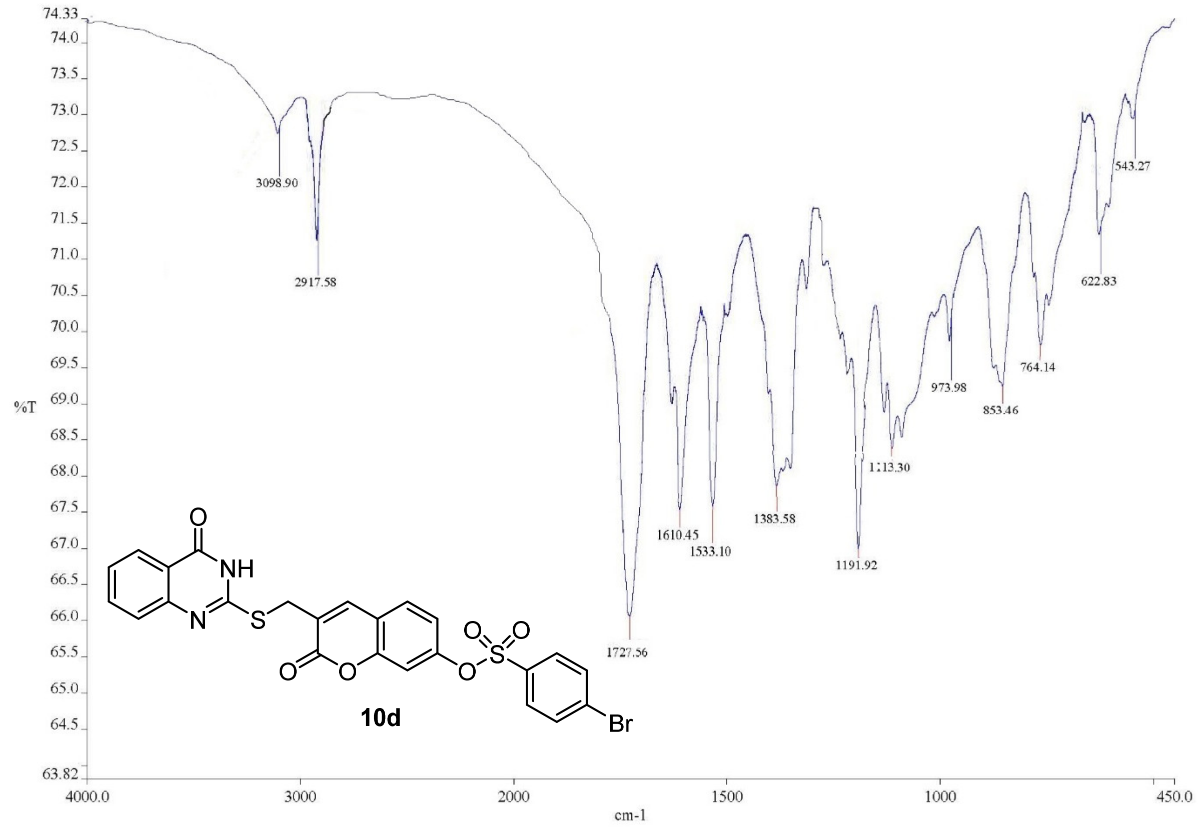

IR spectrum of compound **10d**

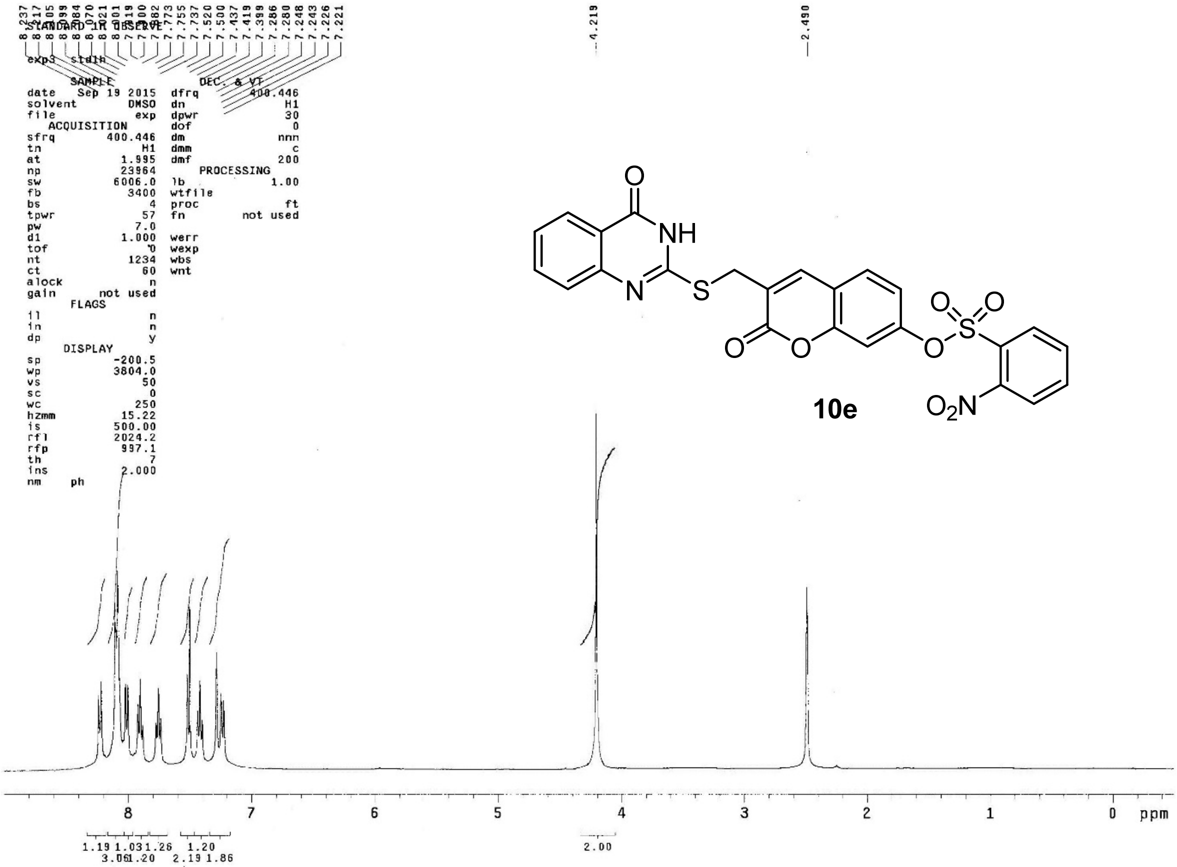

^{1}H NMR spectrum of compound **10e**

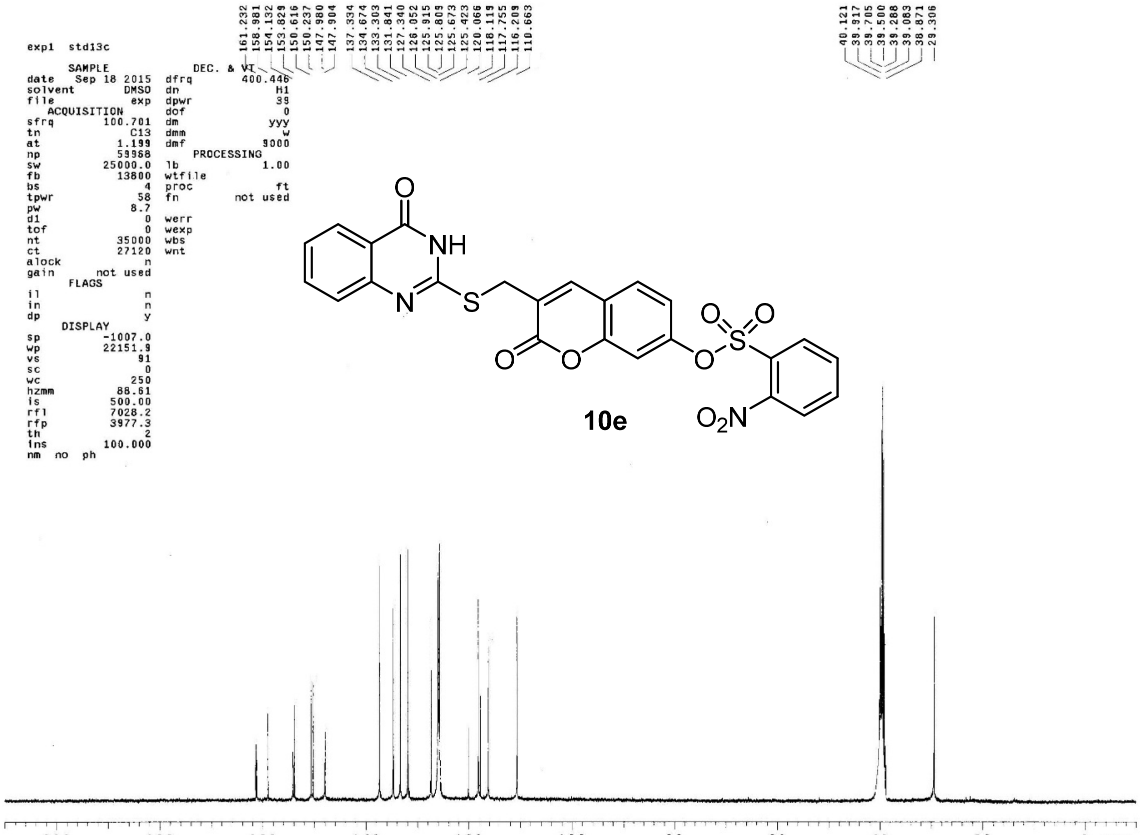

^{13}C NMR spectrum of compound **10e**

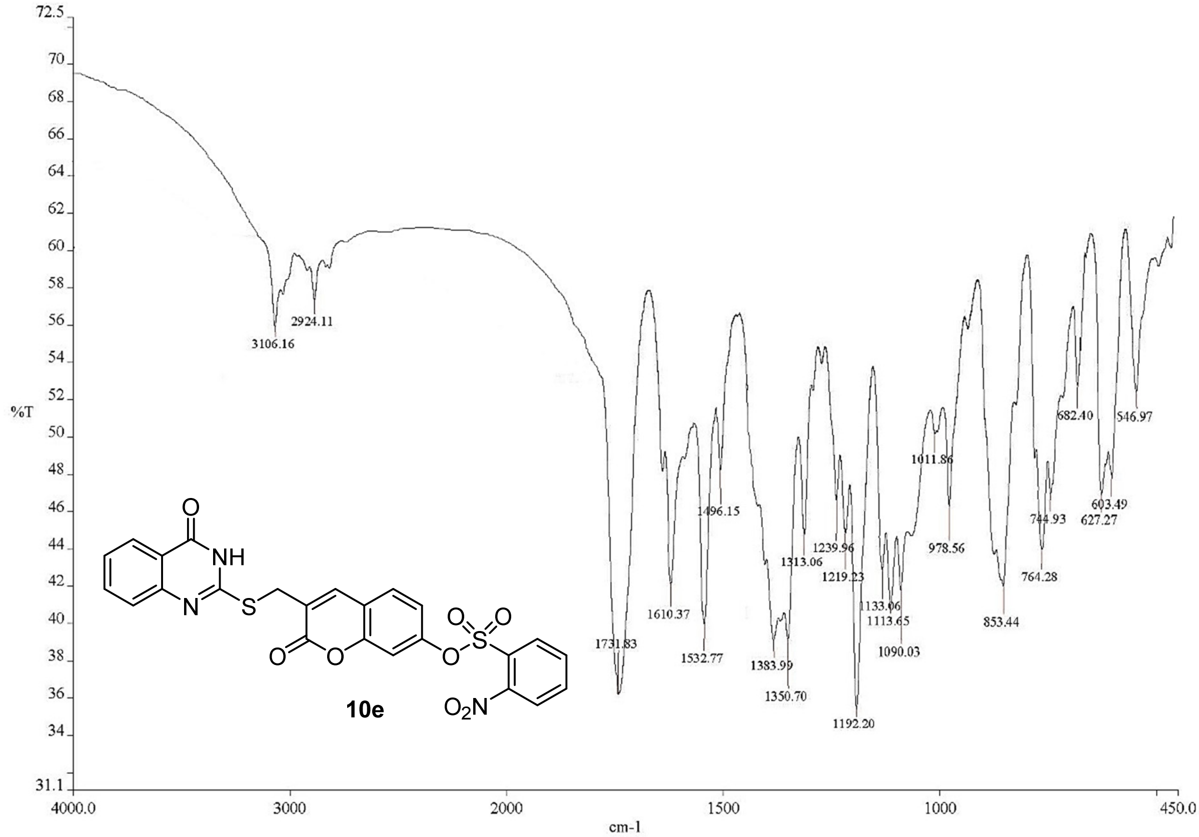

IR spectrum of compound **10e**

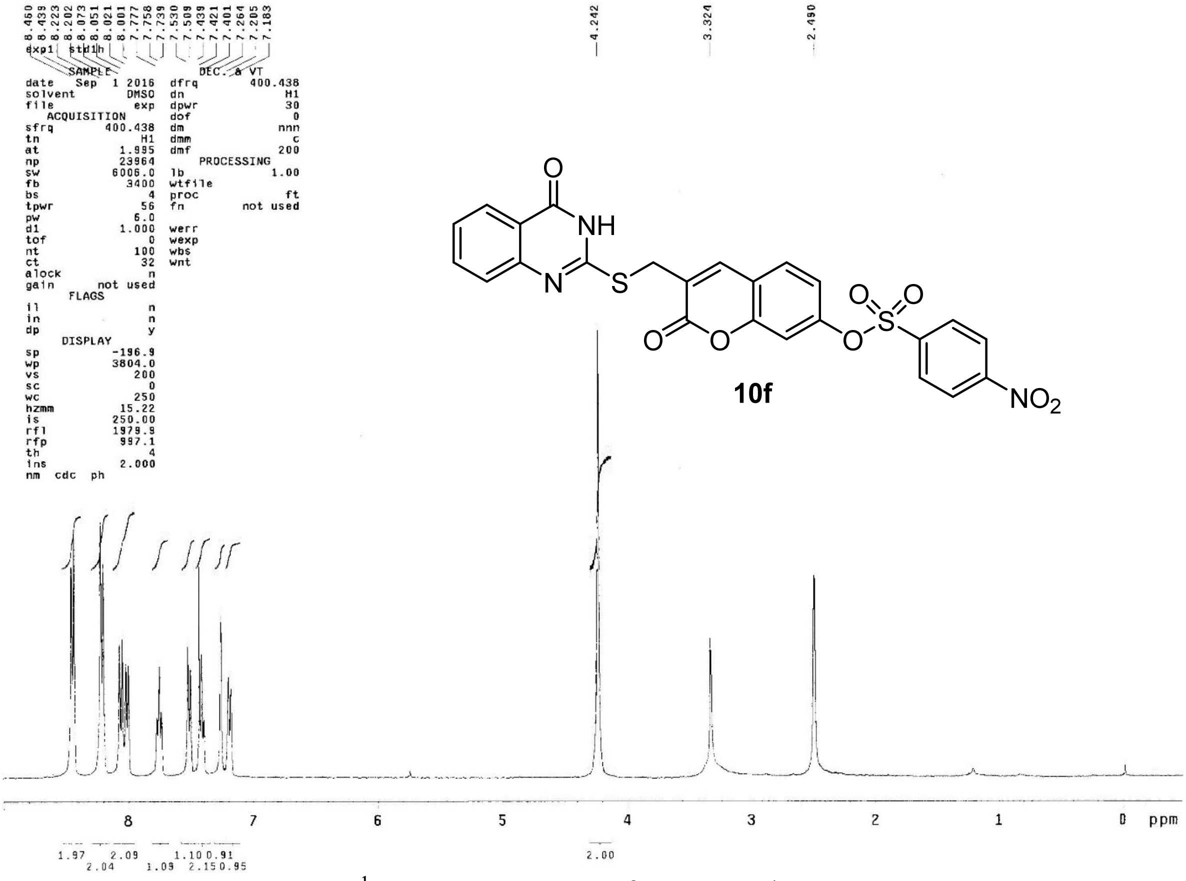

^{1}H NMR spectrum of compound **10f**

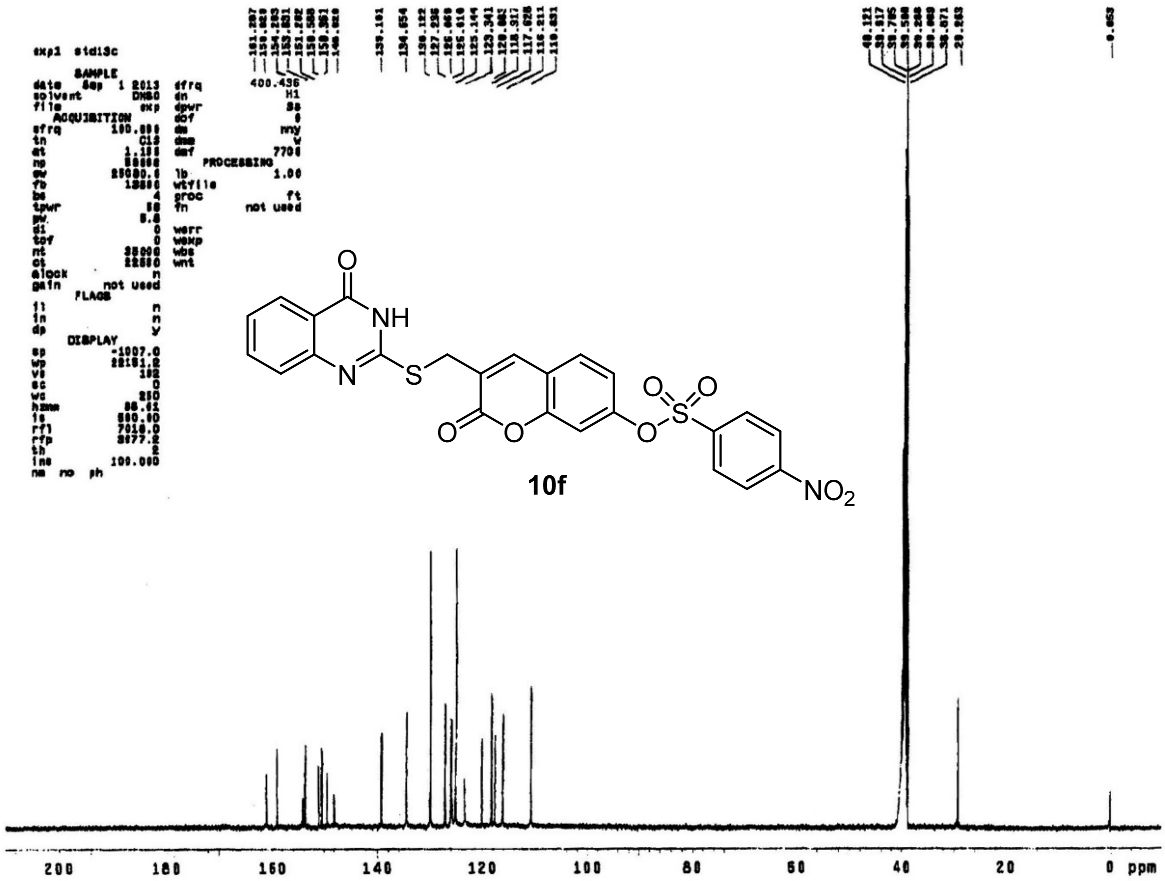

^{13}C NMR spectrum of compound **10f**

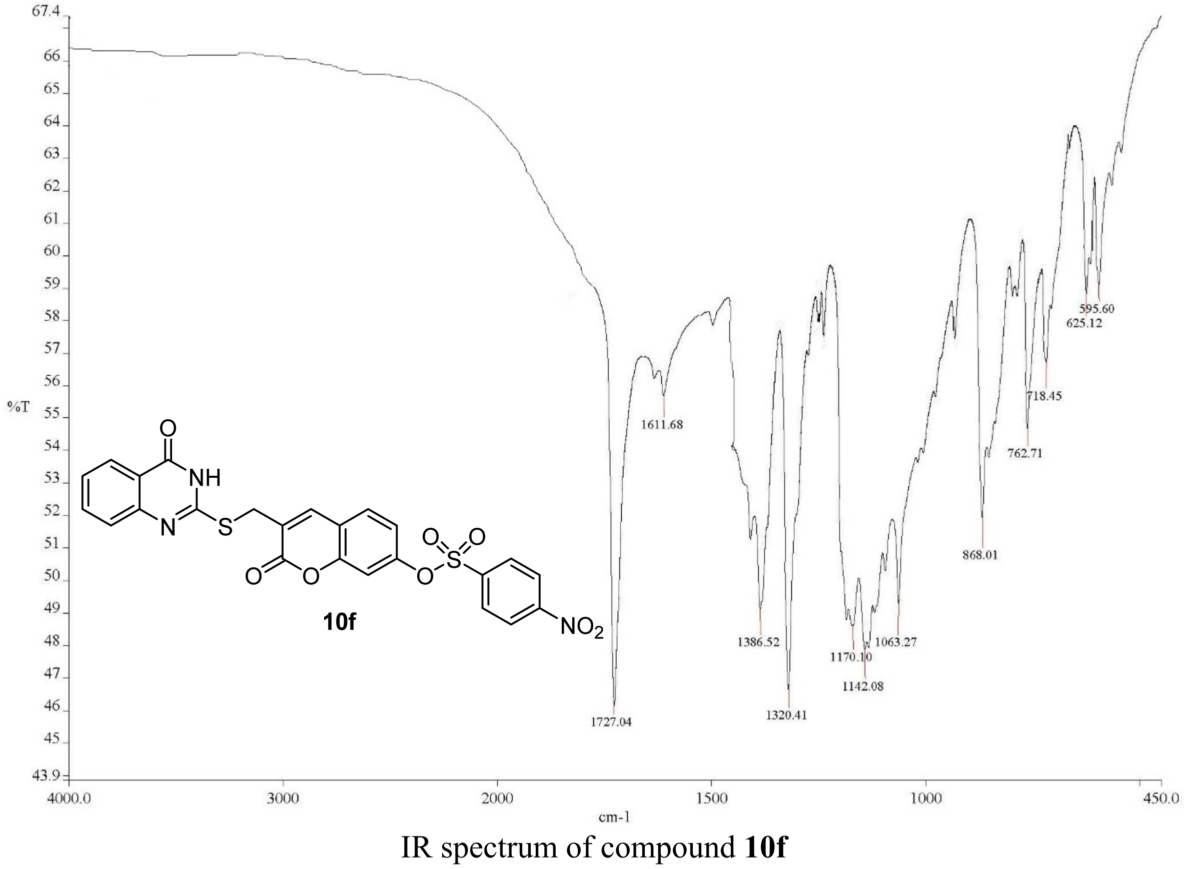

IR spectrum of compound **10f**

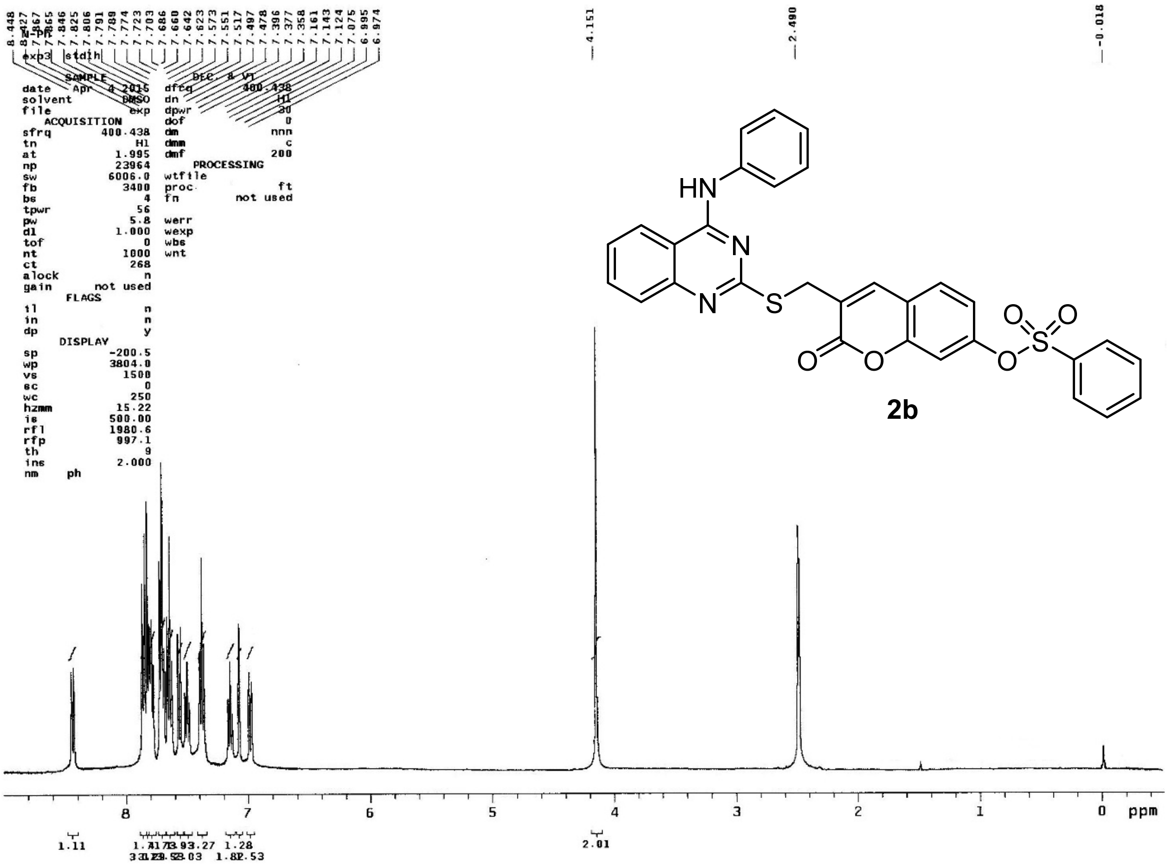

^{1}H NMR spectrum of compound **2b**

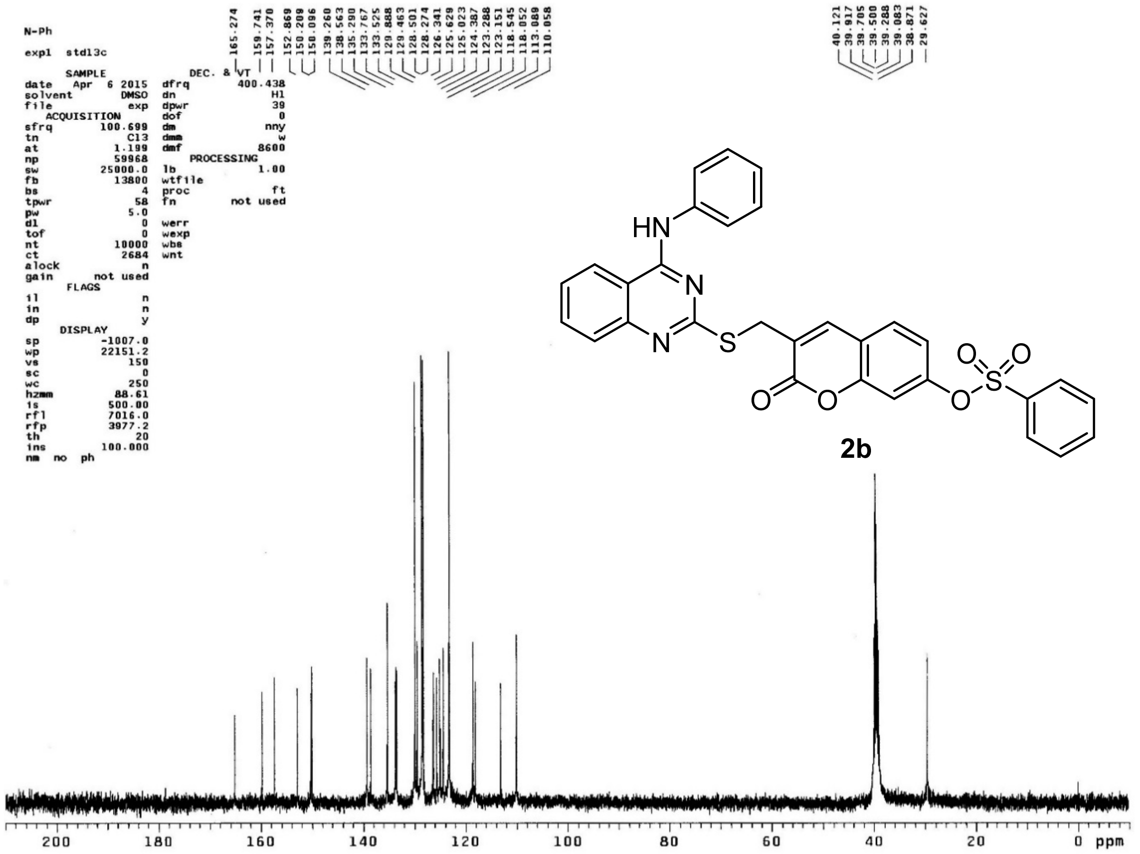

^{13}C NMR spectrum of compound **2b**

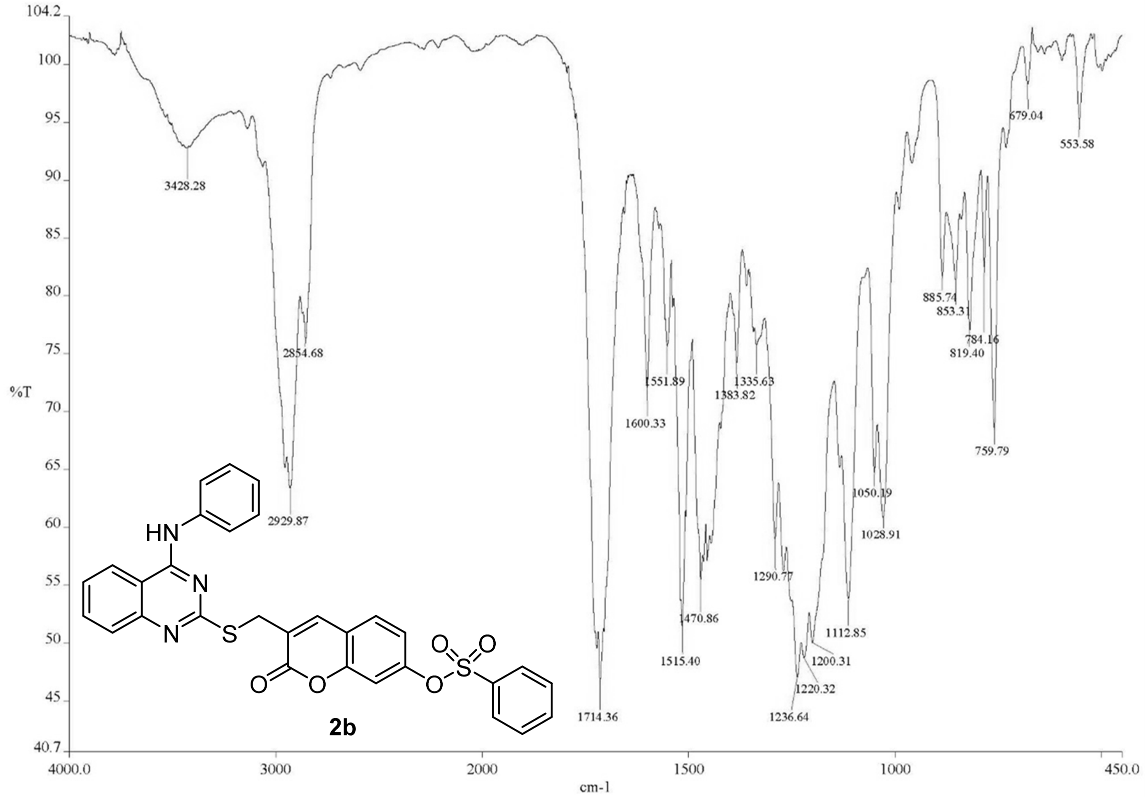

IR spectrum of compound **2b**

HPLC chromatograms for traces of final compounds

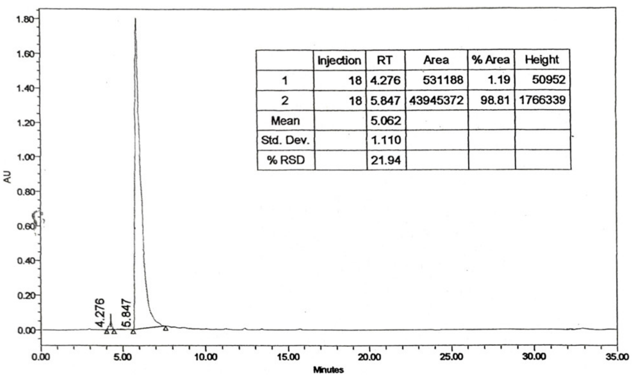

	Injection	RT	Area	% Area	Height
1	18	4.276	531188	1.19	50952
2	18	5.847	43945372	98.81	1766339
Mean		5.062			
Std. Dev.		1.110			
% RSD		21.94			

10a

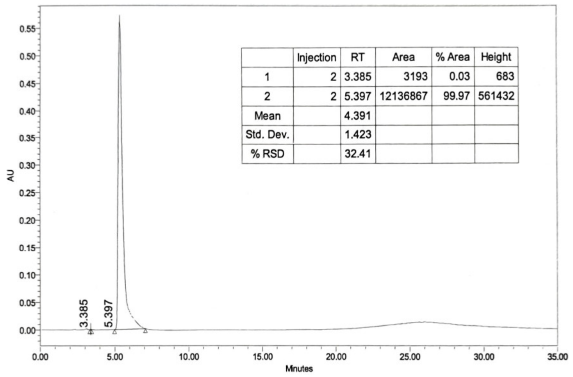

	Injection	RT	Area	% Area	Height
1	2	3.385	3193	0.03	683
2	2	5.397	12136867	99.97	561432
Mean		4.391			
Std. Dev.		1.423			
% RSD		32.41			

10b

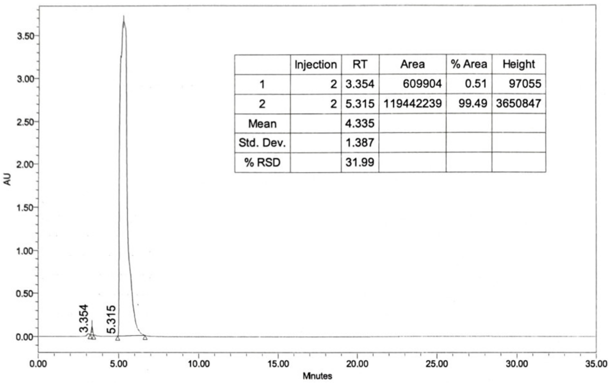

	Injection	RT	Area	% Area	Height
1	2	3.354	609904	0.51	97055
2	2	5.315	119442239	99.49	3650847
Mean		4.335			
Std. Dev.		1.387			
% RSD		31.99			

10c

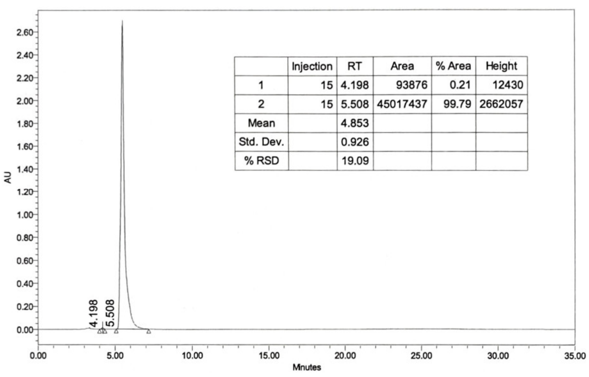

	Injection	RT	Area	% Area	Height
1	15	4.198	93876	0.21	12430
2	15	5.508	45017437	99.79	2662057
Mean		4.853			
Std. Dev.		0.926			
% RSD		19.09			

10d

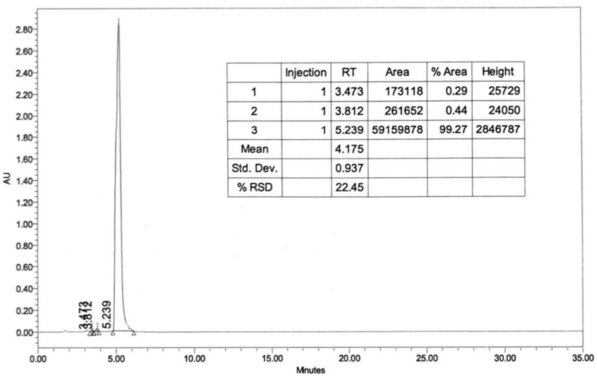

	Injection	RT	Area	% Area	Height
1	1	3.473	173118	0.29	25729
2	1	3.812	261652	0.44	24050
3	1	5.239	59159878	99.27	2846787
Mean		4.175			
Std. Dev.		0.937			
% RSD		22.45			

10e

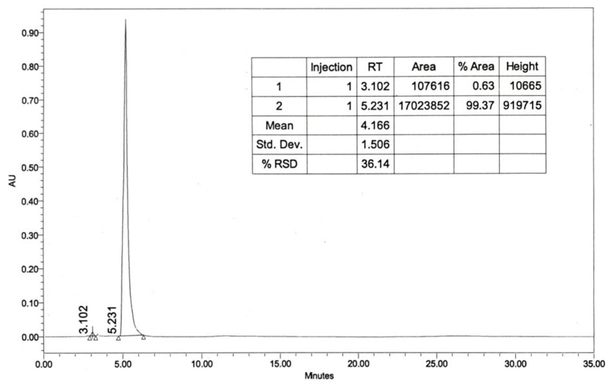

	Injection	RT	Area	% Area	Height
1	1	3.102	107616	0.63	10665
2	1	5.231	17023852	99.37	919715
Mean		4.166			
Std. Dev.		1.506			
% RSD		36.14			

10f

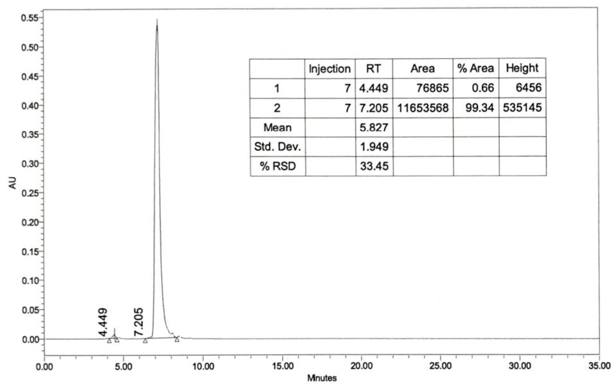

	Injection	RT	Area	% Area	Height
1	7	4.449	76865	0.66	6456
2	7	7.205	11653568	99.34	535145
Mean		5.827			
Std. Dev.		1.949			
% RSD		33.45			

2b
